# Non-ablative dermal remodeling lasers in acquired dermal macular hyperpigmentation: A scoping review

**DOI:** 10.1371/journal.pone.0359552

**Published:** 2026-09-28

**Authors:** Virgilio Blandon, Halyma Gomez, Erick Correa, Miguel Borge, Maria-Jose Altamirano, Alessandro Alvarado, Taylys Leyton

**Affiliations:** 1 Department of Dermatology, Universidad Americana (UAM), Managua, Nicaragua; 2 Department of Dermatology, Universidad de Ciencias Médicas y Enfermería "Dr. Julio Briceño Dávila", Managua, Nicaragua; 3 School of Medicine, Universidad Americana (UAM), Managua, Nicaragua; Faculty of Medical Sciences of Minas Gerais, BRAZIL

## Abstract

**Background:**

Acquired dermal macular hyperpigmentation (ADMH) encompasses a group of challenging pigmentary disorders characterized by dermal melanophages and variable lichenoid interface inflammation. Non-ablative dermal remodeling lasers (NADRL), which target water rather than melanin, have been proposed as a potential therapeutic option through neocollagenesis and modulation of the dermal microenvironment. However, the evidence base for these devices in ADMH has not been systematically characterized.

**Objective:**

To map and characterize the available evidence on NADRL for the treatment of ADMH, including study designs, laser parameters, clinical outcomes, and safety profiles, and to identify knowledge gaps.

**Methods:**

This scoping review followed the methodological frameworks of Arksey and O'Malley and Levac et al., and was reported according to PRISMA-ScR guidelines. A comprehensive search was conducted on June 2, 2026, across PubMed/MEDLINE, LILACS, SciELO, DOAJ, Cochrane Central, and clinical trial registries, supplemented by citation tracking. Studies evaluating NADRL (980–1940 nm) in patients with ADMH were included. Two reviewers independently screened records and extracted data. A narrative synthesis and risk of bias assessment using Cochrane RoB 2 and JBI checklists were performed.

**Results:**

Of 193 records identified through electronic database searches, seven studies met inclusion criteria; citation tracking of relevant reviews and included studies did not identify any additional eligible records. These comprised two randomized controlled trials, three retrospective case series, and two case reports, comprising 31 unique patients. The 1550 nm erbium-doped fractional laser was the most frequently investigated device. Two RCTs showed no clinical or histopathological improvement, whereas observational studies reported favorable outcomes, albeit with significant methodological limitations and concomitant therapies. Laser-induced post-inflammatory hyperpigmentation (PIH) was reported in 23% of patients in one RCT.

**Conclusions:**

Evidence supporting NADRL for ADMH remains limited, heterogeneous, and insufficient to establish independent efficacy. The proposed dermal remodeling mechanism remains largely untested, as most studies evaluated only pigmentary outcomes. Future research should prioritize standardized prospective studies with histological, molecular, and imaging endpoints to determine whether these devices provide true disease modification.

## Introduction

Acquired macular hyperpigmentations of uncertain etiology remain a diagnostic and therapeutic challenge in dermatology. A global consensus statement by Kumarasinghe et al. (2018) grouped several entities with overlapping clinical and histopathological features, including ashy dermatosis (AD), erythema dyschromicum perstans (EDP), lichen planus pigmentosus (LPP), idiopathic eruptive macular pigmentation (IEMP), and Riehl’s melanosis [1]. Clinically, these disorders present as acquired, asymptomatic to mildly pruritic, slate-gray to brown macules with characteristic distribution patterns depending on the entity. Histologically, they share dermal melanophages and variable lichenoid interface inflammation, leading to the umbrella term acquired dermal macular hyperpigmentation (ADMH) [2].

Management of ADMH remains unsatisfactory. Topical agents such as hydroquinone, retinoids, and corticosteroids provide limited and often transient benefit, whereas systemic therapies including clofazimine, dapsone, and isotretinoin are reserved for more extensive disease because of potential adverse effects [3,4]. Recurrence after treatment discontinuation is common, which has led to increasing interest in energy-based devices that can target dermal pigment and underlying inflammation.

Among these, the neodymium-doped yttrium aluminium garnet (Nd) laser has been evaluated primarily at the 1064 nm wavelength in Q-switched or picosecond modalities [5–7]. This wavelength can effectively fragment dermal melanin but is associated with a relevant risk of post-inflammatory hyperpigmentation (PIH), particularly in darker phototypes (Fitzpatrick IV-VI), which are most frequently affected by ADMH [8].

Given that ADMH involves both pigmentary alteration and chronic interface inflammation [1], structural changes within the dermis are likely to contribute to disease persistence. The extracellular matrix (ECM), composed predominantly of collagens such as types I, III, and V, plays a central role in maintaining dermal integrity [9]. In chronic inflammatory skin conditions (such as lichen planus), ECM homeostasis is disrupted by increased matrix metalloproteinase activity and abnormal collagen turnover [9,10]. The lichenoid interface infiltrate characteristic of these conditions produces pro-inflammatory cytokines such as tumour necrosis factor-alpha (TNF-α) and interferon-gamma (IFN-γ) [11], which upregulate MMP-1, MMP-2, and MMP-9, promoting collagen degradation [12]. In line with this, MMP-9 expression has been documented in the lymphocytic infiltrate of lichen planus lesions, suggesting its involvement in basement membrane disruption [11,13]. At the same time, these cytokines suppress collagen-related gene expression, including COL1A1, COL1A2, and COL4A1, leading to an imbalance between degradation and synthesis [12]. Collagen degradation further contributes to inflammation, as matrix fragments act as chemoattractants for immune cells, while intact collagen can inhibit T-cell activity through leukocyte-associated immunoglobulin-like receptor-1 (LAIR-1) [14].

This bidirectional relationship between ECM integrity and inflammation may be relevant to ADMH. A predominantly CD8 + T-cell lichenoid infiltrate induces melanocyte damage and melanin incontinence, resulting in melanophages in the upper dermis [2,15,16]. The resulting matrix alterations may sustain inflammatory signaling and impair the clearance of dermal melanophages. Interventions that restore ECM structure or stimulate collagen remodeling could therefore influence not only dermal architecture but also the local inflammatory milieu [12,14].

In this context, a group of non-ablative infrared lasers has emerged that share the common principle of delivering controlled heat to the dermis while preserving the epidermis. In contrast to the extensively studied 1064 nm wavelength, whose mechanism of action in pigmentary disorders is primarily melanin fragmentation and whose clinical efficacy in ADMH has been modest [5–7], these devices target water as the primary chromophore, such as Nd:YAG (1320 nm-1444 nm) [17–22], diode systems (980 nm and 1410–1470, 1940 nm) [19,23–31], erbium-doped (1540–1565 nm) [25,32,33], and thulium fiber devices (1927 nm) [34]. These lasers have demonstrated a favorable safety and efficacy profile in other dermatologic indications, including neocollagenesis and fibroblast activation in rejuvenation and atrophic acne scars, with histologic confirmation of increased collagen types I, III, and VII [17], and clinical improvements reported across multiple wavelengths [17–29,31–34]; for the 980 nm, and 1444 nm wavelength, evidence is currently limited to preclinical studies [22,23]. Despite mixed clinical results for certain devices in ADMH [35,36], their deeper dermal penetration and lower melanin absorption may reduce the risk of PIH compared with pigment-targeting wavelengths, with direct or indirect evidence across these devices [18,37–39].

In addition to their effects on collagen remodeling, these lasers have been associated with changes in inflammatory skin conditions. Clinical and preclinical studies across multiple wavelengths have reported reductions in inflammatory lesions, erythema, and sebum production, as well as improvements in patient-reported outcomes related to skin inflammation [25,40–52]. These observations suggest that dermal heating, regardless of the specific wavelength, may influence inflammatory pathways, potentially in part through effects on the extracellular matrix and the cellular microenvironment [17,22,23,25,30,41,45,49–54]. While the mechanistic evidence remains incomplete, the anti-inflammatory effects observed with various non-ablative devices further support the hypothesis that these lasers could modulate the inflammatory component of ADMH, beyond their role in dermal remodeling.

Despite this rationale, the evidence on non-ablative dermal remodeling lasers (NADRL) for ADMH has not been systematically characterized. Given this gap, a scoping review represents an appropriate methodological approach. Unlike systematic reviews that address narrowly defined clinical questions, scoping reviews aim to map the available evidence, clarify key concepts, and identify research gaps [55–57]. This approach is particularly suitable for rare conditions such as ADMH, where existing data are likely limited to small studies or case reports. However, this remodeling hypothesis remains unproven in ADMH.

## Methods

### Study design and framework

This scoping review was conducted in accordance with the methodological frameworks of Arksey and O’Malley and Levac et al. and reported following the PRISMA extension for Scoping Reviews (PRISMA-ScR) guidelines [55–57]. Contextualized within a problem-driven research approach [58], the review was motivated by a clinical observation that highlighted the scarcity of published evidence on NADRL in ADMH [59]. The full PRISMA-ScR checklist is provided in S1 Text. The review aimed to systematically identify and map the available literature on NADRL for acquired dermal macular hyperpigmentation (ADMH) and related conditions.

For the purposes of this review, NADRL were defined as infrared devices that use water as the primary chromophore and induce controlled dermal heating, promoting neocollagenesis and fibroblast activation while preserving the epidermis. The 1064 nm Nd wavelength was excluded despite evidence of dermal remodeling effects [60,61], as its primary chromophore is melanin and its mechanism of action in pigmentary disorders is predominantly based on melanin fragmentation. In the context of acquired dermal macular hyperpigmentation, this wavelength is primarily used to target dermal melanophages rather than to induce dermal remodeling [5,7,62], and its inclusion would have introduced substantial conceptual and methodological heterogeneity.

### Objectives and research question

The objective of this review was to map and characterize the existing evidence on NADRL in the management of ADMH. Specifically, the review aimed to describe study designs, laser parameters, and reported clinical, histological, patient-reported, and safety outcomes, as well as to identify gaps in the literature.

The central research question was: What is the volume and nature of evidence regarding the use of NADRL for the treatment of acquired dermal macular hyperpigmentation, and what outcomes have been reported?

### Eligibility criteria

Studies were eligible if they included human participants with a clinical and/or histopathological diagnosis of ADMH and evaluated any NADRL, either as monotherapy or in combination with other treatments. Eligible study designs included primary peer-reviewed research (randomized controlled trials, cohort studies, case series, case reports, and letters with original data). Clinical trial registries were also searched.

Studies were required to report at least one outcome of interest, including clinical improvement, recurrence, histological findings, patient-reported outcomes, or adverse events.

Studies were excluded if they focused on other pigmentary disorders (e.g., melasma, lentigos, nevi, freckles, PIH unrelated to ADMH, poikiloderma, or Addison’s disease), or if they evaluated devices outside the predefined category (e.g., ablative lasers, pigment-targeting lasers, or intense pulsed light). Preclinical studies and preprints were excluded. Reviews, editorials, comments, and conference abstracts were eligible if they reported original primary data; those without primary data were excluded. Articles without accessible full text were also excluded. Publications in English, Spanish, and Portuguese were included, without date restrictions.

### Search strategy

A comprehensive electronic search was conducted on 2 June 2026 across PubMed/MEDLINE, LILACS, SciELO, DOAJ, ClinicalTrials.gov, ClinicalTrials.eu, Cochrane Central Register of Controlled Trials, and the WHO ICTRP to identify studies evaluating NADRL for acquired dermal macular hyperpigmentation and related conditions. This multi-database approach ensured coverage of publications in English, Spanish, and Portuguese, including regional literature not indexed in PubMed, as well as ongoing or completed clinical trials.

In PubMed, search strategies combined controlled vocabulary and free-text terms for the target conditions (ADMH, ashy dermatosis, erythema dyschromicum perstans, lichen planus pigmentosus, idiopathic eruptive macular pigmentation, Riehl's melanosis, and pigmented contact dermatitis) with terms related to NADRL. A comprehensive list of relevant wavelengths (1320 nm, 980 nm, 1410 nm, 1444 nm, 1450 nm, 1470 nm, 1540 nm, 1550 nm, 1565 nm, 1570 nm, 1927 nm, and 1940 nm) was included, along with broader terms such as “non-ablative,” “fractional laser,” “dermal remodeling,” and “water-targeting” to capture studies that did not specify wavelength.

Searches in LILACS, SciELO, and DOAJ were conducted using equivalent terms in Spanish, Portuguese, and English, whereas Cochrane Central was searched using English terms only. Clinical trial registries were searched using condition-specific terms. Citation tracking was also performed to identify additional relevant studies.

No date restrictions were applied, and language was limited to English, Spanish, and Portuguese. All records were imported into Rayyan for deduplication and preliminary screening, followed by manual verification. Full search strategies and results for each database are provided in S1 Table.

### Study selection

After deduplication, which was performed by a single reviewer and subsequently verified by a second reviewer, all records underwent title and abstract screening against the predefined eligibility criteria. The full texts of potentially relevant studies were then assessed for inclusion. Both the title/abstract and full‑text screening phases were conducted independently by two reviewers who were blinded to each other's decisions. Disagreements between the two reviewers were resolved through consultation with a third reviewer, who served as an arbiter to reach consensus. The results of the deduplication, screening, and article inclusion/exclusion process are summarized in Supporting information S2 and S3 Tables, which provide counts of duplicates removed, unique records screened, exclusions at each stage, and final articles included for full-text review.

### Citation tracking

Citation tracking was conducted iteratively to enhance the comprehensiveness of the search. Backward citation searching was performed on all review articles identified through the electronic searches, regardless of their inclusion status, to identify potentially relevant primary studies. In addition, both backward and forward citation tracking were applied to all studies included in the final review.

This process aimed to identify additional eligible primary studies. Citation tracking was conducted by a single reviewer during the prescreening phase, and potentially relevant records were flagged for inclusion in the subsequent blinded screening process. No records identified through citation tracking met the eligibility criteria during prescreening, and therefore none progressed to full-text review. A detailed summary of the citation tracking process is provided in S4 Table.

### Data charting and evidence synthesis

A predefined data extraction template was used to collect the first author and publication year, condition studied, laser device and wavelength, treatment parameters, clinical outcomes, adverse events, and study design for each included study. Data extraction was performed by one reviewer and verified by a second to ensure accuracy and consistency.

Evidence was synthesized descriptively to summarize study characteristics, laser modalities, clinical and safety outcomes, and the quality of the available evidence. Given the heterogeneity of study designs, laser parameters, and outcome measures, a narrative synthesis was considered the most appropriate approach.

### Risk of bias assessment

A descriptive risk of bias assessment was performed to contextualize the mapped literature, following methodological guidance for scoping reviews [55–57]. Design-specific tools were applied: Cochrane RoB 2 for randomized trials (RCT) [63], and JBI checklists for case reports and series [64,65]. Initial assessments were conducted by one reviewer and verified by a second; disagreements were resolved by consensus. Findings were synthesized narratively to highlight methodological patterns across the evidence base.

### Protocol registration

This scoping review was not preregistered. To ensure transparency and reproducibility, all methodological materials are provided as supplementary files, including search strategies, screening decisions, exclusion codes, citation tracking data, risk of bias assessments, and the completed PRISMA-ScR checklist. These materials provide a fully auditable and reproducible review process.

### Timeline

The electronic literature search was completed on 2 June 2026. Title and abstract screening was completed on 15 June 2026, and full‑text screening was completed on 21 June 2026. Following this, citation tracking was conducted during the on 22 June 2026. Data charting of the full‑text excluded studies was completed on 10 July 2026, and the writing of the results section was finalized on 20 July 2026. This timeline reflects the sequential completion of all methodological steps and ensures transparent reporting of the review process.

## Results

### Search results and study selection

A total of 193 records were identified through the electronic database searches. After deduplication, 160 unique records were screened by title and abstract. Of these, 127 records were excluded based on predefined eligibility criteria, and 33 records were selected for full-text review. Following full-text assessment, 26 records were excluded, leaving seven studies that met all inclusion criteria and were included in the final synthesis.

Citation tracking was performed to complement the electronic searches. Backward citation searching of 15 relevant review articles (one additional review could not be retrieved) and of the seven included studies yielded 914 references (831 from reviews and 83 from the included studies). Forward citation searching of the seven included studies, conducted on June 22, 2026, identified an additional 160 references. In total, citation tracking identified 1,074 records. During pre-screening, none of these records met the eligibility criteria, as they addressed different laser wavelengths, focused on hyperpigmentation disorders outside the predefined scope, or were not primary studies. Consequently, no records from citation tracking advanced to title and abstract screening, dual independent review, or full-text assessment.

Thus, while the total number of records initially identified from all sources was 1,267 (comprising 193 from electronic databases and 1,074 from citation tracking), only the 193 database records were carried forward for deduplication and formal screening. The complete study selection process is presented in the PRISMA-ScR flow diagram (Fig 1).

**Fig 1:**
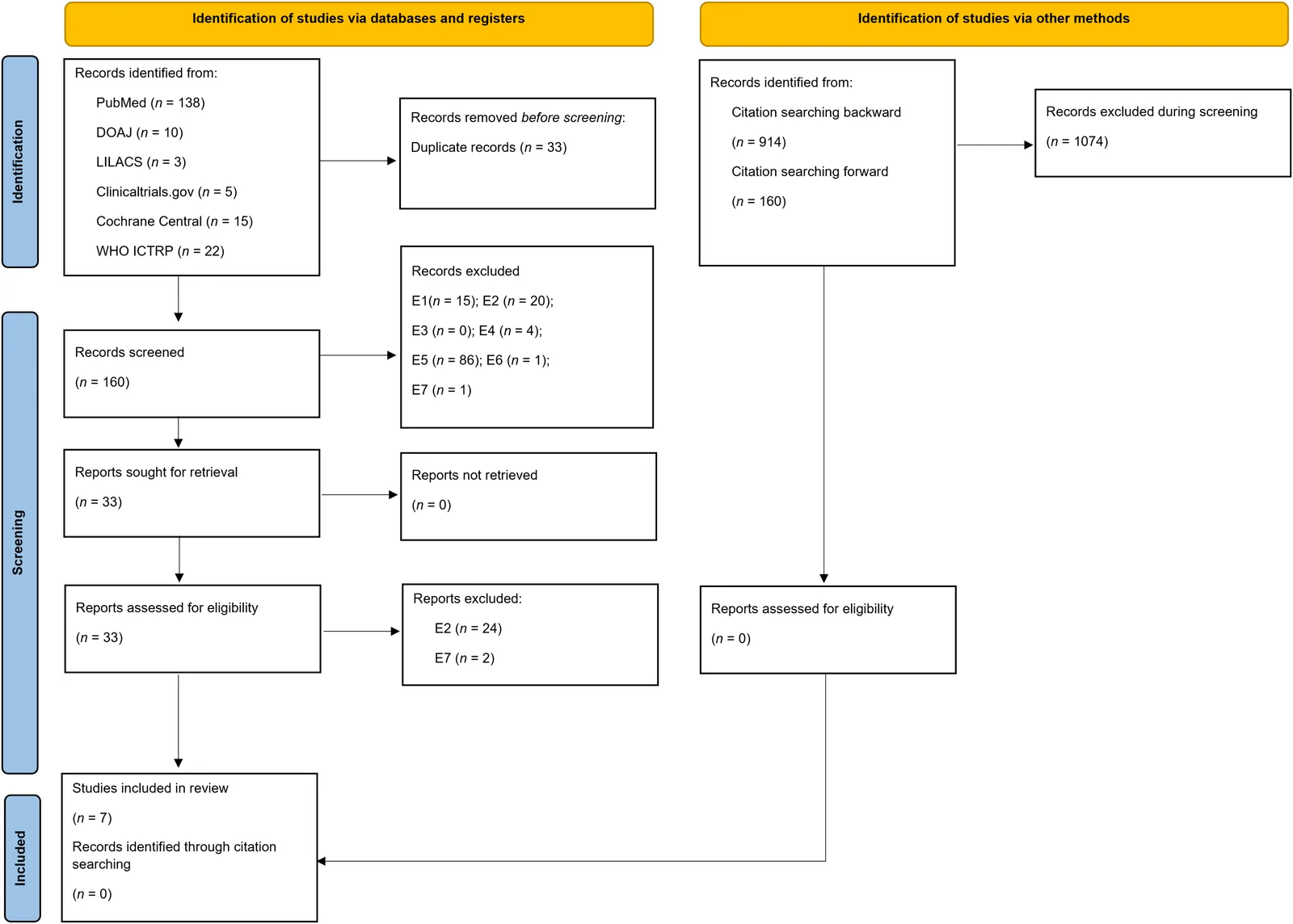
PRISMA-ScR flow diagram of study selection. Note. This figure illustrates the identification, screening, eligibility assessment, and inclusion of studies evaluating NADRL for acquired dermal macular hyperpigmentation (ADMH) and related conditions. The selection process reflects the scarcity of evidence in ADMH and the predominantly exploratory nature of the available literature.

### Characteristics of included studies

Seven studies met the inclusion criteria for this scoping review, encompassing a spectrum of study designs: two observer‑blinded split‑lesion RCTs [35,36], three retrospective case series [52,66,67], and two single‑patient case reports [68,69]. The included studies were published between 2012 and 2024, reflecting a slow but growing interest in energy‑based devices for these challenging conditions.

Although two of the early RCTs included patients with PIH and Becker's nevus [35,36], only the subset of patients meeting the operational definition of ADMH were considered for this review. It is important to note that these two RCTs are derived from the same parent cohort of patients [35,36], with the studies presenting different analytical perspectives: one focusing on clinical efficacy [35], and the other on histopathological findings [36].

Across the seven included studies, a total of 37 patients with confirmed ADMH were identified based on the predefined eligibility criteria, reflecting only the subset of patients meeting inclusion criteria within studies that often enrolled mixed diagnostic populations. However, this cumulative figure is misleading: a substantial proportion of the evidence derives from small observational reports, including two single-patient case reports [68,69], one case series contributing a single eligible patient from a two-patient cohort [66], two additional case series contributing nine and eleven patients, respectively [52,67], and the remaining two studies contributing eight and six eligible patients from RCTs [35,36]. Furthermore, these two trials share a subset of six patients with ashy dermatosis, reducing the total number of unique patients to 31. This distribution underscores the limited and fragmented nature of the evidence base, in which certain ADMH subtypes, such as linear LPP or Riehl’s melanosis, rely on evidence from a single study.

Across the included studies, Fitzpatrick skin types ranged from II to V, representing the phototypes commonly affected by acquired dermal macular hyperpigmentation and those at increased risk of PIH. The 1550 nm erbium-doped fractional laser was the most frequently investigated device, followed by the 1927 nm fractional thulium fiber laser. One study employed a multimodal protocol incorporating a 1570 nm erbium-glass laser in combination with ablative CO_2_ laser resurfacing, precluding evaluation of the independent effect of the non-ablative component. Laser treatment parameters varied substantially, including pulse energy, treatment density, number of passes, and treatment sessions, reflecting the lack of standardized protocols and the exploratory nature of the available evidence.

Most studies combined laser therapy with adjunctive treatments, including topical tacrolimus, cysteamine, intralesional polydeoxyribonucleotide, or intermittent depigmenting regimens, limiting attribution of clinical outcomes to laser treatment alone. Only two studies evaluated laser monotherapy, although participants had previously failed multiple therapeutic approaches. Outcome assessment was heterogeneous and included physician-rated clinical scales, objective pigmentation measurements, histopathological evaluation, and patient-reported satisfaction. Follow-up ranged from 3 to 9.1 months, with limited and inconsistent reporting of long-term outcomes and recurrence.

Of the seven studies included, three reported direct associations with Solta Medical, the manufacturer of Fraxel laser systems. Kroon et al. and Wind et al. stated that laser devices and consumables were provided by B&Co Laser Medico and Solta Medical Inc. exclusively for research purposes, without involvement in study design or data interpretation, while Wolfshohl et al. included an author (Paul M. Friedman, MD) who serves on the Solta, Inc. Advisory Board, despite no reported external funding. Notably, the two industry-supported RCTs (Kroon and Wind) found no clinical benefit of the 1550 nm laser for ADMH or PIH, whereas the only well-documented case report with industry-affiliated authorship (Wolfshohl) described favorable outcomes, albeit in combination with topical tacrolimus.

Critical appraisal of the included studies identified several methodological limitations. Diagnostic criteria for acquired dermal macular hyperpigmentation were inconsistent: only three studies confirmed the diagnosis histopathologically before treatment, whereas the remainder relied on clinical assessment or included heterogeneous subtypes without stratification [66,68,69]. None distinguished between active inflammatory lesions and quiescent pigmentary sequelae, despite the potential influence of disease activity on treatment response and the risk of PIH. Moreover, no study was specifically designed to evaluate dermal remodeling as the primary mechanism underlying clinical improvement. Instead, reported benefits were largely attributed to melanin fragmentation and transepidermal elimination, providing only indirect support for the dermal remodeling hypothesis explored in this review.

Substantial clinical and methodological heterogeneity, including differences in laser wavelengths, treatment parameters, treatment intervals, adjunctive therapies, outcome measures, and follow-up duration, precluded direct comparison across studies. A summary of the key characteristics and principal findings of the included studies is presented in Table 1, whereas comprehensive study details, including laser parameters, adjunctive therapies, quantitative efficacy outcomes, study limitations, and funding sources, are provided in S5 Table.

**Table 1:** Summary of included studies.

Author, Year	Study Design	Laser Device (Wavelength)	Population (ADMH Subtype, *n*)	Principal Findings
Kroon MW, et al., 2012	RCT, split-lesion, observer-blinded	1550 nm Er:glass (Fraxel re:store)	EDP & PIH (*n* = 8* EDP; *n* = 6 PIH)	No clinical improvement; PhGA and melanin index showed no significant differences. Laser-induced PIH in 23%.
Wind BS, et al., 2012	RCT, split-lesion, observer-blinded (histological substudy)	1550 nm Er:glass (Fraxel re:store)	AD (*n* = 6*) & PIH (*n* = 4)	No histopathological improvement; no reduction in dermal melanophages; no MTZ evidence at 3 months.
Wolfshohl JA, et al., 2016	Case report	1550 nm Er:glass (Fraxel)	EDP (*n* = 1, F, Fitzpatrick IV)	>75% improvement at 3 months; maintained at 8 months.
Kim SM, et al., 2020	Case series	1927 nm TFL (LASEMD™)	Riehl's melanosis (*n* = 9, Fitzpatrick III–IV)	DPASI: 9.55 → 5.25; 67% achieved 51–75% improvement; no PIH reported.
Jung CJ, et al., 2023	Case report (within a case series of two patients)	1927 nm TFL (Fraxel SR)	Linear LPP (*n* = 1, F, Fitzpatrick III)	Improvement in hyperpigmentation after 7 sessions; mild residual atrophy.
Murray TN, et al., 2023	Case report	1550 nm Er:glass (Fraxel DUAL)	LPP-like drug reaction (*n* = 1, F, Fitzpatrick V)	>80% improvement at 3 months after 7 treatments.
Garg S, et al., 2024	Case series	1570 nm Er:glass + CO_2_ (sequential protocol; 1570 nm used only for resistant areas)	ADMH (LPP, EDP, AD, Riehl's) (*n* = 11, Fitzpatrick III–IV)	72.6% achieved >75% improvement; mean physician score: 3.81 ± 1.33.

Note. AD, ashy dermatosis; ADMH, acquired dermal macular hyperpigmentation; DPASI, dermal pigmentation area and severity index; EDP, erythema dyschromicum perstans; Er:glass, erbium-doped glass; F, female; FU, follow-up; LPP, lichen planus pigmentosus; MTZ, microscopic treatment zone; PDRN, polydeoxyribonucleotide; PhGA, Physician's Global Assessment; PIH, post-inflammatory hyperpigmentation; RCT, randomized controlled trial; RoB, risk of bias; TFL, thulium fiber laser. *: Kroon et al. and Wind et al. share the same cohort of six patients with ashy dermatosis; after accounting for this overlap, the total number of unique patients across all studies is 31. Three studies reported industry affiliation with Solta Medical (Kroon, Wind, Wolfshohl), although funders had no role in study design or data interpretation.

### Risk of bias appraisal

Overall, study quality was heterogeneous. The RCTs demonstrated moderate internal validity, with limitations primarily related to incomplete blinding and the absence of pre-specified analysis plans or protocol registration. One RCT was judged to have a high risk of bias due to substantial unexplained missing outcome data (28% attrition).

Case series and case reports provided valuable descriptive insights into patient characteristics, interventions, and outcomes, but were inherently limited in their ability to support causal inference or generalizable conclusions. Only one case report was assessed as having a low risk of bias, reflecting complete reporting across all appraisal domains, although its single-case design limits external validity.

Across the evidence base, recurrent methodological limitations included small sample sizes, heterogeneous intervention protocols, retrospective designs, lack of consecutive patient inclusion, and reliance on subjective outcome measures. In line with the exploratory objective of this scoping review, no studies were excluded based on risk of bias. A summary of the assessments is presented in Table 2.

**Table 2:** Risk of bias assessment of included studies.

Author, Year	Study Design	Assessment Tool	Overall Risk of Bias	Key Methodological Limitations
Kroon, 2012	RCT, observer-blinded split-lesion	Cochrane RoB 2	Some concerns	Randomization method and allocation concealment not specified; participants and treating physicians unblinded (observer-blinded only); no pre-specified analysis plan or protocol registration.
Wind, 2012	RCT, observer-blinded split-lesion (histological substudy)	Cochrane RoB 2	High	Substantial missing outcome data (7/25 patients, 28%) without explanation or sensitivity analysis; comparison between non-ablative and ablative lasers not randomized (different patient populations); no protocol registration.
Wolfshohl, 2016	Case report	JBI Checklist for Case Reports	Low	Complete reporting across all JBI domains; single-patient design inherently limits external validity. Industry affiliation (Solta Advisory Board) noted but no reported influence.
Kim, 2020	Case series	JBI Checklist for Case Series	High	Retrospective design; variable treatment protocols (3–7 sessions); confounders (oral prednisolone in 5/9 patients); objective measures (melanin index) only for 2 patients; histology for 1 patient.
Jung, 2023	Case series (n = 2)	JBI Checklist for Case Series	High	Consecutive/complete inclusion not explicitly stated; combination therapy (tacrolimus + PDRN) prevents isolating laser effect; no post-treatment histology; statistical analysis not applicable.
Murray, 2023	Case report	JBI Checklist for Case Reports	High	Adverse events not explicitly described; single-patient design; combination with cysteamine confounds laser effect; short follow-up (3 months).
Garg, 2024	Case series	JBI Checklist for Case Series	High	Retrospective design; consecutive/complete inclusion not explicitly stated; no control group; multi-laser sequential protocol prevents isolating 1570 nm effect; subjective assessments only (no objective measurements); no statistical analysis within diagnostic subgroups.

Note. RCT, randomized controlled trial; RoB, risk of bias; JBI, Joanna Briggs Institute; PDRN, polydeoxyribonucleotide. Risk of bias was assessed using Cochrane RoB 2 for RCTs (randomization, deviations, missing data, outcome measurement, selective reporting) and JBI checklists for case reports (demographics, history, diagnostics, intervention, outcomes, adverse events, lessons) and case series (inclusion, condition measurement, consecutive inclusion, demographics, clinical data, outcomes, site information, analysis). No studies were excluded based on risk of bias, consistent with the exploratory scope of this review.

### Synthesis of findings

The available evidence on NADRL for acquired dermal macular hyperpigmentation remains limited and heterogeneous. Clinical outcomes varied according to study design and intervention context. The two RCTs evaluating 1550 nm erbium-doped fractional laser reported no significant clinical or histopathological improvement in ashy dermatosis or PIH [35,36], whereas smaller observational studies described favorable responses with 1550 nm and 1927 nm devices [52,66–69]. These positive outcomes were reported primarily in case reports and retrospective series, where interpretation was limited by small sample sizes and the frequent use of concomitant therapies.

Across studies, the contribution of laser treatment alone could not be consistently determined. Most interventions incorporated adjunctive treatments, including topical immunomodulators, depigmenting agents, or regenerative therapies [35,36,52,66–69]. Histological assessments were infrequently performed and did not demonstrate consistent evidence of dermal remodeling as a treatment endpoint [35,36,52]. Reported improvements were mainly evaluated through pigmentation scores or clinical assessments rather than objective measures of dermal structural changes [35,52,66–69].

Three included studies reported an association with Solta Medical, the manufacturer of Fraxel laser systems [35,36,68]. Although the reported industry involvement did not result in exclusion or an increased risk of bias in the appraisal process, the current evidence base remains concentrated around a limited number of devices and investigators. The overall findings indicate that NADRL remain investigational for ADMH, with insufficient evidence to establish their independent efficacy or to support dermal remodeling as the primary mechanism underlying clinical improvement.

## Discussion

### Principal findings and current state of evidence

This scoping review identified a limited and heterogeneous evidence base for NADRL in ADMH. Although clinical improvement has been reported, current evidence does not establish consistent therapeutic efficacy. RCTs have not demonstrated clear clinical or histopathological benefit [35,36], whereas favorable outcomes have predominantly originated from uncontrolled observational studies with important methodological limitations [52,66–69].

A major limitation of the current evidence is the inability to determine the independent contribution of laser therapy. The frequent use of adjunctive treatments, heterogeneous protocols, lack of control groups, and limited objective assessment of dermal remodeling substantially restrict causal interpretation [35,36,52,66–69]. Moreover, a notable disconnect exists between the proposed therapeutic mechanism and the outcomes used to evaluate treatment efficacy. Although the biological rationale for dermal remodeling in ADMH is plausible [9,10,12,14], most studies evaluated only pigmentary outcomes rather than biological or structural markers of dermal remodeling, leaving the proposed mechanism largely untested [35,52,66–69].

It is also noteworthy that the available evidence is heavily concentrated on the 1550 nm erbium-doped fractional laser, which accounts for the majority of published data in ADMH. Despite the theoretical rationale and the inclusion of other NADRL in our search strategy, including Nd:YAG 1320 nm, diode systems (980 nm, 1410–1470 nm, 1940 nm), erbium-doped devices (1540 nm, 1565 nm, 1570 nm), and thulium fiber lasers (1927 nm), no published studies evaluating these devices in ADMH met our eligibility criteria. While this gap may reflect a lack of clinical investigation or publication bias, it is also plausible that the predominance of the 1550 nm wavelength in the literature is partly influenced by industry support, as three of the included studies reported associations with the manufacturer of the Fraxel laser system [35,36,68]. Whether this commercial interest has shaped the research agenda, rather than the therapeutic potential of other devices, remains an open question. Nonetheless, this concentration of evidence underscores that the current knowledge base cannot be generalized to the broader category of water-targeting non-ablative lasers.

Overall, NADRL should be considered investigational in ADMH until higher-quality evidence clarifies their independent efficacy and their potential role in modifying disease-related dermal alterations. Future studies should prioritize standardized laser protocols, histological endpoints, objective outcome measures, and controlled designs capable of separating laser effects from concomitant therapies.

### Interpreting the discrepancy between randomized trials and observational evidence

The evidence for laser therapy in ADMH presents an apparent paradox, with RCTs reporting no significant benefit while multiple observational reports describe marked clinical improvement. However, this discrepancy is likely explained by differences in study design, patient selection, disease stage, and therapeutic strategy rather than true contradiction.

Importantly, the two available RCTs are not fully independent, as they share a subset of patients, meaning that the negative evidence derives from a limited cohort rather than from separate confirmatory studies. Moreover, both trials evaluated a 1550 nm non-ablative fractional laser using relatively conservative parameters in patients with active, histologically confirmed inflammatory disease, treated concurrently with topical depigmenting agents (hydroquinone, tretinoin, and low-potency triamcinolone) [35,36]. In this context, adjunctive corticosteroids were used intermittently and primarily to mitigate treatment-related irritation, rather than as a targeted strategy to control the underlying immune-mediated process [70,71].

In contrast, the positive observational reports describe a fundamentally different therapeutic context. These studies often involve patients with long-standing or stable pigmentary sequelae and employ a more deliberate integration of immunomodulation. For instance, Kim et al. administered systemic corticosteroids prior to laser therapy to control active inflammation, effectively separating the inflammatory phase from the pigmentary phase before intervention [52]. Similarly, Jung et al. and Wolfshohl et al. combined laser treatment with continuous topical tacrolimus in a chronic setting, directly targeting T-cell-mediated pathways implicated in lichenoid disorders [66,68]. Notably, Wolfshohl et al. employed the same 1550 nm erbium-doped fractional laser used in the negative RCTs, with comparable energy and coverage settings (5–9 mJ vs. 15 mJ; 14% coverage in both), yet achieved marked clinical improvement [68]. This suggests that the key difference may lie in the concomitant use of continuous topical tacrolimus and application during a stable disease phase rather than active inflammation; a hypothesis that extrapolates the recognized importance of disease stage when using lasers in ADMH [72]. Although disease activity was not systematically evaluated in the included studies, this interpretation is consistent with the available observations and should be considered hypothesis-generating, warranting prospective evaluation, precluding attribution to the laser alone.

Taken together, the apparent discrepancy between RCTs and observational reports likely reflects the heterogeneity of ADMH. The current evidence does not definitively exclude a role for laser therapy but suggests that its effectiveness may be context-dependent. Future studies should aim to stratify patients according to disease activity, incorporate rational immunomodulatory approaches, and compare different laser modalities in order to better define optimal treatment strategies.

### Biological rationale: Beyond pigment clearance toward dermal disease modulation

The rationale for using NADRL in acquired dermal macular hyperpigmentation challenges the traditional view of ADMH as a disorder primarily defined by residual dermal pigment. These conditions involve persistent alterations in the inflammatory microenvironment and dermal stroma, suggesting that melanophages may represent a consequence of an ongoing dermal process rather than the sole therapeutic target [16,73]. Histopathological evidence demonstrates progressive accumulation of dermal melanophages with increasing disease duration [74], while recent molecular studies have identified dermal fibroblast-derived mediators such as neuregulin-1 (NRG1) and dickkopf-1 (DKK1) as biomarkers associated with disease severity and dermal pigmentation activity [75]. Together, these findings support the concept of ADMH as an active dermal disorder rather than a static pigmentary condition. However, whether NADRL can influence these disease-related dermal alterations remains largely unknown, as available studies have primarily evaluated pigmentary outcomes without directly assessing structural or molecular changes within the dermis.

A similar therapeutic concept has emerged with platelet-rich plasma (PRP) in lichen planus pigmentosus. In the initial prospective pilot study, PRP-associated clinical improvement was attributed to several potential mechanisms, including modulation of melanogenesis, enhanced dermal pigment clearance, immunoregulatory effects, and a more speculative regenerative effect based on collagen remodeling reported in other dermatologic settings [76]. However, these mechanisms were not directly evaluated in LPP, and the proposed regenerative role remained hypothetical.

The subsequent split-face RCT illustrates a methodological challenge also encountered in many laser studies [77]. Although PRP was proposed to influence the dermal microenvironment, the study assessed outcomes mainly through pigmentary measures, while both treatment sides received topical hydroquinone. Consequently, the trial primarily evaluated whether PRP provided additional depigmenting benefit rather than whether it modified dermal alterations underlying pigmentation. Moreover, no histological or imaging endpoints were included to evaluate potential regenerative effects. Thus, despite a biologically plausible mechanism, the study did not directly assess the pathway it was hypothesized to influence.

Importantly, mechanistic evaluation of dermal remodeling after energy-based therapies is technically feasible and has already been demonstrated using multiple histological and molecular approaches. Experimental studies of non-ablative Nd:YAG 1320 nm laser have incorporated serial skin biopsies with quantitative assessment of collagen remodeling, elastin dynamics, and extracellular matrix reorganization [17], whereas studies of microneedling radiofrequency have additionally evaluated molecular markers associated with pigmentary regulation, including CD44 and bFGF [78]. Although these findings do not establish definitive disease modification, they highlight the importance of integrating biological endpoints to determine whether energy-based therapies act solely through pigment clearance or also through modulation of disease-related dermal pathways.

Future studies evaluating NADRL in ADMH should therefore incorporate mechanistic outcomes, including histopathology, molecular biomarkers, or non-invasive imaging modalities such as reflectance confocal microscopy, which can provide in vivo longitudinal assessment of dermal remodeling and pigment architecture [75,79,80]. Without these biological endpoints, the current literature cannot determine whether NADRL influence the pathogenic processes sustaining ADMH or simply improves its visible manifestations.

### Safety considerations and risk of post-inflammatory hyperpigmentation

Although non-ablative dermal remodeling lasers theoretically offer a safety advantage in ADMH because they primarily target water rather than epidermal melanin [81,82], this benefit has not been demonstrated clinically in this review. As discussed above, treatment safety may be influenced not only by laser parameters but also by disease activity at the time of intervention [70,72]. This may explain why PIH occurred in the RCT by Kroon et al. despite conservative treatment settings [35]. In contrast, favorable outcomes in observational studies were generally reported after inflammatory control had been achieved or in combination with sustained immunomodulatory therapy [52,66,68,69]. Although speculative, this hypothesis provides a biologically plausible explanation for the inconsistent safety outcomes reported across studies. Similarly, the efficacy of these devices as independent treatments has not been established, as most positive reports involved combination regimens.

The current evidence is further limited by inconsistent safety reporting. Adverse events and PIH were not systematically assessed, objective pigment measurements were infrequently used, and no study specifically evaluated Fitzpatrick skin types V-VI, despite these populations being at the greatest risk of pigmentary complications [1,2]. Of note, only one study included a single Fitzpatrick V patient [69], and none included Fitzpatrick VI. Consequently, the safety profile of NADRL in ADMH remains incompletely characterized. Future prospective studies should incorporate standardized adverse-event reporting, objective pigment assessment, adequate representation of darker phototypes, and stratification by disease activity to determine whether inflammatory control influences treatment safety and efficacy.

### Methodological limitations and implications for future research

The current evidence base for NADRL in ADMH is limited by important methodological constraints that should guide future research priorities. The available randomized evidence derives from a single patient cohort, while most supportive data originate from small observational studies with heterogeneous protocols and limited control of confounding factors [35,52,66–69]**.** Future investigations should prioritize multicenter prospective controlled trials with standardized treatment protocols, adequate sample sizes, blinded outcome assessment when feasible, and prespecified analyses to isolate the treatment effect attributable to laser therapy.

A major challenge is the heterogeneity of ADMH populations and the inconsistent assessment of disease activity. Future studies should prospectively stratify patients according to predefined disease activity categories, as this variable may modify both efficacy and safety. Integration of molecular biomarkers may improve characterization of disease activity and provide objective mechanistic endpoints for evaluating treatment response, although their predictive value requires prospective validation [75,78]. In addition, adequate inclusion of Fitzpatrick skin types V-VI is essential to improve the generalizability of efficacy and safety findings in populations most affected by ADMH [1,2].

Outcome assessment also requires standardization. Current studies use heterogeneous clinical scales and rarely incorporate objective pigment measurements. Future trials should include validated outcomes such as DPASI, complemented by melanin index, reflectance spectroscopy, or colorimetric analysis [83], and should evaluate long-term outcomes to determine durability and recurrence [84,85]. Establishing a core outcome set for ADMH studies would facilitate comparison between trials and future evidence synthesis.

Future studies should incorporate mechanistic endpoints, including histology [17], molecular biomarkers [75,78], and non-invasive imaging modalities such as reflectance confocal microscopy [79,80], to determine whether NADRL produce true disease modification rather than isolated pigment clearance. Such approaches would enable direct evaluation of the proposed therapeutic mechanism by assessing changes in collagen architecture, extracellular matrix remodeling, melanophage burden, and inflammatory pathways after treatment.

Finally, the safety profile of NADRL in ADMH remains incompletely characterized. Standardized reporting of adverse events, objective assessment of PIH, and prespecified analyses according to Fitzpatrick skin type are needed. Additionally, comparative studies evaluating different laser wavelengths and treatment strategies, including rational combinations with immunomodulatory therapies according to disease phase, may clarify whether specific approaches provide superior efficacy or safety.

Overall, NADRL research in ADMH remains at an early stage. Progress will require a transition from small descriptive studies toward collaborative, prospective, biologically informed trials capable of determining whether these devices provide true disease modification or only transient pigmentary improvement.

### Limitations of the scoping review

This review was limited by the small number of available studies and substantial heterogeneity in study designs, laser parameters, treatment protocols, outcome measures, and follow-up periods, which prevented quantitative synthesis. The predominance of small observational studies and case reports limits the strength of conclusions regarding NADRL efficacy and safety in ADMH. Although a comprehensive search strategy was performed, some relevant studies may have been missed, particularly those not indexed in the searched databases, published in other languages, or unavailable in full text. Furthermore, because this scoping review was conducted within a problem-driven research framework, the research question and conceptual boundaries were informed by an identified clinical knowledge gap, which may have influenced the scope of the evidence mapped despite the use of predefined eligibility criteria and a systematic search strategy. Additionally, the review was not prospectively registered, which may represent a methodological limitation despite the use of predefined eligibility criteria and extraction procedures.

## Conclusion

Evidence supporting non-ablative dermal remodeling lasers (NADRL) for acquired dermal macular hyperpigmentation (ADMH) remains limited, heterogeneous, and insufficient to establish their independent efficacy. The frequent use of concomitant therapies in positive reports, combined with the lack of controlled studies isolating the laser effect, further underscores that any observed benefits cannot be attributed to the laser alone. Although dermal remodeling has been proposed as their principal therapeutic mechanism, existing studies have evaluated predominantly pigmentary outcomes rather than biological or structural markers of disease modification. Consequently, whether these devices influence the underlying dermal pathology of ADMH remains unknown. Future research should prioritize standardized prospective studies incorporating objective clinical, histological, molecular, and imaging endpoints to determine whether NADRL produce true disease modification beyond pigment clearance.

## Supporting information

S1 Appendix Complete screening process. (XLSX)

S1 Text PRISMA-ScR checklist. (DOCX)

S2 Text Completed risk of bias assessment questionnaires. (DOCX)

S1 Table Search strategy and results across all information sources (electronic databases and citation tracking). Note: The search was conducted on June 2, 2026, across PubMed, SciELO, LILACS, DOAJ, Cochrane central, ClinicalTrials.gov, ClinicalTrials.eu, and the WHO ICTRP. Search strings combined a broad set of terms for pigmentary disorders (e.g., ashy dermatosis, erythema dyschromicum perstans, lichen planus pigmentosus, Riehl's melanosis, and acquired dermal macular hyperpigmentation) with terms for laser therapies (covering various modalities and wavelengths), adapted to the language and syntax of each database. For citation tracking: backward searching was performed on all review articles identified from the primary database searches, without exception. Additionally, both backward and forward searching were performed on all articles that were ultimately included after the title/abstract and full-text screening process. The total number of records retrieved from all sources was 1,267.
(DOCX)

S2 Table Summary of article inclusion, deduplication, and exclusion during the selection process. Note. This table summarizes the full selection process for studies included in the review. It reports the total number of records retrieved before deduplication, the number of duplicate and unique records identified, and the distribution of exclusions at the title and abstract screening stage. Exclusion reasons are provided according to predefined categories, including population outside the predefined conditions, review/editorial articles, conference abstracts, animal studies, inadequate interventions, insufficient data, lack of full-text availability, and inappropriate study design. Articles meeting eligibility criteria at this stage were included for full-text review.
(DOCX)

S3 Table Full-text review inclusion and exclusion. This table summarizes the outcomes of the full-text screening phase. It reports the number of articles excluded after full-text assessment along with the reasons for exclusion, based on predefined eligibility criteria. The table provides a quantitative overview of inclusion and exclusion decisions at the full-text screening stage.
(DOCX)

S4 Table Summary of backward and forward citation searches across databases. Note. This table reports the results of backward and forward citation searches performed on selected source articles to identify additional studies potentially eligible for inclusion. For each round, the table provides the source article ID, date of the search (forward searches only), search type (backward or forward), total results retrieved and number of articles meeting inclusion criteria.
(DOCX)

S5 Table Comprehensive study details of included articles on laser therapy for acquired dermal macular hyperpigmentation (ADMH). Note. Summary of included studies on energy-based devices for acquired dermal macular hyperpigmentation (ADMH). Studies are organized chronologically by year of publication. Abbreviations: AD, ashy dermatosis; ADMH, acquired dermal macular hyperpigmentation; BN, Becker's nevus; DPASI, dermal pigmentation area and severity index; EDP, erythema dyschromicum perstans; Er:YAG, erbium-doped yttrium aluminum garnet; H&E, hematoxylin and eosin staining; FLT, fractional laser therapy; IPL, intense pulsed light; LPP, lichen planus pigmentosus; MTZ, microscopic treatment zone; Nd:YAG, neodymium-doped yttrium aluminum garnet; PDRN, polydeoxyribonucleotide; PDL, pulsed dye laser; PhGA, Physician's Global Assessment; PIH, postinflammatory hyperpigmentation; QS, quality-switched; TFL, thulium fiber laser; VAS, visual analogue scale. Note: The asterisk (*) indicates that Kroon et al. and Wind et al. share a subset of six patients with ashy dermatosis (erythema dyschromicum perstans); after accounting for this overlap, the total number of unique patients across all studies is 31 (rather than the cumulative total of 37).
(DOCX)
