# Supplementary material for: Non-ablative dermal remodeling lasers in acquired dermal macular hyperpigmentation: A scoping review
Source: PLoS One. 2026 Sep 28;21(9):e0359552. doi: 10.1371/journal.pone.0359552 (PMC13618957; doi:10.1371/journal.pone.0359552)
Supplement: Supplementary file 2 [file pone.0359552.s003.docx]

**JBI Critical Appraisal Checklist for Case Reports**

**Reviewers:** Virgilio Blandon; Halyma Gomez
**Date:** June 2026
**Author:** Murray TN, Rajanala S, Friedman PM
**Year:** 2023
**Record Number:** Pubmed-62

| **Question** | **Yes** | **No** | **Unclear** | **Not applicable** |
| --- | --- | --- | --- | --- |
| 1. Were patient's demographic characteristics clearly described? | X |  |  |  |
| 2. Was the patient's history clearly described and presented as a timeline? | X |  |  |  |
| 3. Was the current clinical condition of the patient on presentation clearly described? | X |  |  |  |
| 4. Were diagnostic tests or assessment methods and the results clearly described? | X |  |  |  |
| 5. Was the intervention(s) or treatment procedure(s) clearly described? | X |  |  |  |
| 6. Was the post-intervention clinical condition clearly described? | X |  |  |  |
| 7. Were adverse events (harms) or unanticipated events identified and described? |  | X |  |  |
| 8. Does the case report provide takeaway lessons? | X |  |  |  |

**Overall appraisal:** ☑ Include ☐ Exclude ☐ Seek further info

**Comments:** This case report provides a well-documented description of a patient with lichen planus pigmentosus-like drug reaction treated with a combination of 1550-nm fractional laser and topical cysteamine cream. The report clearly describes patient demographics, clinical history with a clear timeline, diagnostic methods including histopathology, detailed treatment parameters, and post-intervention outcomes with clinical photographs. The takeaway lessons are clinically relevant. However, adverse events are not explicitly described, though none were reported. The report provides valuable hypothesis-generating evidence for a condition with limited therapeutic options.

**JBI Critical Appraisal Checklist for Case Reports**

**Reviewers:** Virgilio Blandon; Halyma Gomez
**Date:** June 2026
**Author:** Wolfshohl et al.
**Year:** 2017
**Record Number:** Pubmed-11

| **Question** | **Yes** | **No** | **Unclear** | **Not applicable** |
| --- | --- | --- | --- | --- |
| 1. Were patient’s demographic characteristics clearly described? | X |  |  |  |
| 2. Was the patient’s history clearly described and presented as a timeline? | X |  |  |  |
| 3. Was the current clinical condition of the patient on presentation clearly described? | X |  |  |  |
| 4. Were diagnostic tests or assessment methods and the results clearly described? | X |  |  |  |
| 5. Was the intervention(s) or treatment procedure(s) clearly described? | X |  |  |  |
| 6. Was the post-intervention clinical condition clearly described? | X |  |  |  |
| 7. Were adverse events (harms) or unanticipated events identified and described? | X |  |  |  |
| 8. Does the case report provide takeaway lessons? | X |  |  |  |

**Overall appraisal:** ☑ Include ☐ Exclude ☐ Seek further info

**Comments:** This case report provides a detailed and well-documented description of a patient with biopsy-confirmed erythema dyschromicum perstans treated with a combination of 1550-nm fractional laser and topical tacrolimus. All JBI criteria are adequately met. The report clearly describes patient demographics, clinical history, diagnostic methods, intervention parameters, post-treatment outcomes, and adverse events. The takeaway lessons are clinically relevant and support the need for prospective studies. The case provides valuable hypothesis-generating evidence for a condition with limited therapeutic options.

**JBI Critical Appraisal Checklist for Case Series**

**Reviewers:** Virgilio Blandon; Halyma Gomez
**Date:** June 2026
**Author:** Garg S, Vashisht KR, Garg D, Oberoi B, Sharma G
**Year:** 2024
**Record Number:** Pubmed-16

| **Question** | **Yes** | **No** | **Unclear** | **Not applicable** |
| --- | --- | --- | --- | --- |
| 1. Were there clear criteria for inclusion in the case series? | X |  |  |  |
| 2. Was the condition measured in a standard, reliable way for all participants included in the case series? | X |  |  |  |
| 3. Were valid methods used for identification of the condition for all participants included in the case series? | X |  |  |  |
| 4. Did the case series have consecutive inclusion of participants? |  | X |  |  |
| 5. Did the case series have complete inclusion of participants? |  | X |  |  |
| 6. Was there clear reporting of the demographics of the participants in the study? | X |  |  |  |
| 7. Was there clear reporting of clinical information of the participants? | X |  |  |  |
| 8. Were the outcomes or follow up results of cases clearly reported? | X |  |  |  |
| 9. Was there clear reporting of the presenting site(s)/clinic(s) demographic information? | X |  |  |  |
| 10. Was statistical analysis appropriate? | X |  |  |  |

**Overall appraisal:** ☑ Include ☐ Exclude ☐ Seek further info

**Comments:** This retrospective case series reports on 122 Indian patients with various dermal pigmentary conditions treated with sequential laser protocols. The study provides clear inclusion criteria, detailed demographic and clinical information, and appropriate statistical analysis comparing outcomes across diagnostic groups (ANOVA, Tukey's HSD). However, consecutive or complete inclusion of participants is not explicitly stated, and the retrospective design with subjective outcome assessment (physician and patient scales) limits the strength of conclusions. The study provides valuable real-world evidence for sequential laser approaches in skin of color and contributes hypothesis-generating data for future prospective research.

**JBI Critical Appraisal Checklist for Case Series**

**Reviewers:** Virgilio Blandon; Halyma Gomez
**Date:** June 2026
**Author:** Jung CJ, Lee WJ, Won CH, Lee MW, Chang SE
**Year:** 2023
**Record Number:** Pubmed-49

| **Question** | **Yes** | **No** | **Unclear** | **Not applicable** |
| --- | --- | --- | --- | --- |
| 1. Were there clear criteria for inclusion in the case series? | X |  |  |  |
| 2. Was the condition measured in a standard, reliable way for all participants included in the case series? | X |  |  |  |
| 3. Were valid methods used for identification of the condition for all participants included in the case series? | X |  |  |  |
| 4. Did the case series have consecutive inclusion of participants? |  | X |  |  |
| 5. Did the case series have complete inclusion of participants? |  | X |  |  |
| 6. Was there clear reporting of the demographics of the participants in the study? | X |  |  |  |
| 7. Was there clear reporting of clinical information of the participants? | X |  |  |  |
| 8. Were the outcomes or follow up results of cases clearly reported? | X |  |  |  |
| 9. Was there clear reporting of the presenting site(s)/clinic(s) demographic information? | X |  |  |  |
| 10. Was statistical analysis appropriate? |  |  |  | X |

**Overall appraisal:** ☑ Include ☐ Exclude ☐ Seek further info

**Comments:** This case series reports two cases of linear lichen planus pigmentosus treated with fractional lasers and polydeoxyribonucleotide injections. The report provides detailed clinical and histopathological information for both cases, with clear demographic data and outcome reporting. However, the study does not describe consecutive or complete inclusion of participants, and statistical analysis is not applicable for a two-case series. The report provides valuable hypothesis-generating evidence for a rare condition with limited treatment options.

**JBI Critical Appraisal Checklist for Case Series**

**Reviewers:** Virgilio Blandon; Halyma Gomez
**Date:** June 2026
**Author:** Kim SM, Hwang S, Almurayshid A, Park MY, Oh SH
**Year:** 2020
**Record Number:** Pubmed-8

| **Question** | **Yes** | **No** | **Unclear** | **Not applicable** |
| --- | --- | --- | --- | --- |
| 1. Were there clear criteria for inclusion in the case series? | X |  |  |  |
| 2. Was the condition measured in a standard, reliable way for all participants included in the case series? | X |  |  |  |
| 3. Were valid methods used for identification of the condition for all participants included in the case series? | X |  |  |  |
| 4. Did the case series have consecutive inclusion of participants? |  | X |  |  |
| 5. Did the case series have complete inclusion of participants? |  | X |  |  |
| 6. Was there clear reporting of the demographics of the participants in the study? | X |  |  |  |
| 7. Was there clear reporting of clinical information of the participants? | X |  |  |  |
| 8. Were the outcomes or follow up results of cases clearly reported? | X |  |  |  |
| 9. Was there clear reporting of the presenting site(s)/clinic(s) demographic information? | X |  |  |  |
| 10. Was statistical analysis appropriate? | X |  |  |  |

**Overall appraisal:** ☑ Include ☐ Exclude ☐ Seek further info

**Comments:** This retrospective case series provides valuable evidence on the use of 1927-nm fractional thulium fiber laser for Riehl's melanosis. The study includes nine patients with clear inclusion criteria and uses validated outcome measures (DPASI, quartile grading scale, melanin index). However, consecutive inclusion is not explicitly stated, and retrospective design with small sample size limits generalizability. Outcomes are well-reported with objective measurements and follow-up data. The study contributes hypothesis-generating evidence for a condition with limited therapeutic options.

Revised Cochrane risk-of-bias tool for randomized trials (RoB 2)

TEMPLATE FOR COMPLETION

Edited by Julian PT Higgins, Jelena Savović, Matthew J Page, Jonathan AC Sterne
on behalf of the RoB2 Development Group

**Version of 22 August 2019**

Reviewers: Virgilio Blandon; Halyma Gomez

The development of the RoB 2 tool was supported by the MRC Network of Hubs for Trials Methodology Research (MR/L004933/2- N61), with the support of the host MRC ConDuCT-II Hub (Collaboration and innovation for Difficult and Complex randomised controlled Trials In Invasive procedures - MR/K025643/1), by MRC research grant MR/M025209/1, and by a grant from The Cochrane Collaboration.

This work is licensed under a [Creative Commons Attribution-NonCommercial-NoDerivatives 4.0 International License](http://creativecommons.org/licenses/by-nc-nd/4.0/).

**Study details**

| **Reference** | Kroon MW, Wind BS, Meesters AA, Wolkerstorfer A, van der Veen JP, Bos JD, Van der Wal AC, Beek JF. Non-ablative 1550 nm fractional laser therapy not effective for erythema dyschromicum perstans and postinflammatory hyperpigmentation: a pilot study. J Dermatolog Treat. 2012 Oct;23(5):339-44. doi: 10.3109/09546634.2011.565016. Epub 2011 Jul 14. PMID: 21756152. |
| --- | --- |

**Study design**

| □ | Individually-randomized parallel-group trial |
| --- | --- |
| □ | Cluster-randomized parallel-group trial |
| X | Individually randomized cross-over (or other matched) trial |

**For the purposes of this assessment, the interventions being compared are defined as**

| Experimental: | - Laser: Fraxel re:store (Solta Medical), 1550 nm, 5 sessions at 3-week intervals, 8 passes per session, 15 mJ per microbeam, coverage 14-20% depending on skin type. - Topical bleaching: Hydroquinone 5%, tretinoin 0.05%, and triamcinolone acetonide 0.1% cream, applied once daily during the second and third week after each laser session. | Comparator: | - **Topical bleaching:** Hydroquinone 5%, tretinoin 0.05%, and triamcinolone acetonide 0.1% cream, applied once daily during the second and third week after each laser session (on the control area). |
| --- | --- | --- | --- |

| **Specify which outcome is being assessed for risk of bias** | Improvement of hyperpigmentation assessed by: Physician's Global Assessment (PhGA) – Blinded dermatologist evaluation using photographs, scored on a 0–6 scale (0 = total clearance, 6 = worsening of hyperpigmentation). This is the main outcome parameter as stated in the methods. |
| --- | --- |

| **Specify the numerical result being assessed.** In case of multiple alternative analyses being presented, specify the numeric result (e.g. RR = 1.52 (95% CI 0.83 to 2.77) and/or a reference (e.g. to a table, figure or paragraph) that uniquely defines the result being assessed. | Result: No significant difference between the laser-treated and control regions.  Numerical result reported:  *"In the erythema dyschromicum perstans group, the results of the blinded dermatologist assessment (PhGA) did not significantly differ between both test regions"* (Results section, paragraph 3).  *"In the PIH group, PhGA did not significantly differ between both test regions"* (Results section, paragraph 4).  No specific p-value, effect estimate, or confidence interval is reported for the PhGA comparison. The study only states that the difference was not statistically significant. |
| --- | --- |

**Is the review team’s aim for this result…?**

| X | to assess the effect of *assignment to intervention* (the ‘intention-to-treat’ effect) |
| --- | --- |
| □ | to assess the effect of *adhering to intervention* (the ‘per-protocol’ effect) |

**If the aim is to assess the effect of *adhering to intervention***, select the deviations from intended intervention that should be addressed (at least one must be checked):

□	occurrence of non-protocol interventions

□	failures in implementing the intervention that could have affected the outcome

□	non-adherence to their assigned intervention by trial participants

**Which of the following sources were obtained to help inform the risk-of-bias assessment? (tick as many as apply)**

X	Journal article(s) with results of the trial

□	Trial protocol

□	Statistical analysis plan (SAP)

□	Non-commercial trial registry record (e.g. ClinicalTrials.gov record)

□	Company-owned trial registry record (e.g. GSK Clinical Study Register record)

□	“Grey literature” (e.g. unpublished thesis)

□	Conference abstract(s) about the trial

□	Regulatory document (e.g. Clinical Study Report, Drug Approval Package)

□	Research ethics application

□	Grant database summary (e.g. NIH RePORTER or Research Councils UK Gateway to Research)

□	Personal communication with trialist

□	Personal communication with the sponsor

## Risk of bias assessment

Responses underlined in green are potential markers for low risk of bias, and responses in red are potential markers for a risk of bias. Where questions relate only to sign posts to other questions, no formatting is used.

**Domain 1: Risk of bias arising from the randomization process**

| **Signalling questions** | **Comments** | **Response options** |
| --- | --- | --- |
| **1.1 Was the allocation sequence random?** | The study by Kroon et al. (2012) states:  *"In each patient, two similar square test regions of ~10 cm² of affected skin were* ***randomized*** *to receive either FLT in combination with intermittent topical bleaching or the same intermittent regimen of topical bleaching alone"* (Methods).  However, it does not specify:  How the randomization sequence was generated (e.g., computer-generated, random number table).  How allocation was concealed (e.g., sealed opaque envelopes, central randomization, sequentially numbered containers). | PY |
| **1.2 Was the allocation sequence concealed until participants were enrolled and assigned to interventions?** |  | PN |
| **1.3 Did baseline differences between intervention groups suggest a problem with the randomization process?** | Baseline characteristics are inherently balanced because the same patient provides both the experimental and control regions. The two test regions were described as "two similar square test regions of ~10 cm²", indicating that they were chosen to be comparable. The randomization was applied to the regions within each patient, not to separate groups of patients. | N |
| **Risk-of-bias judgement** | The randomization process is likely adequate, but the lack of methodological detail (especially regarding allocation concealment) prevents a definitive "Low risk" judgement. | Some concerns |
| Optional: What is the predicted direction of bias arising from the randomization process? |  | Towards null |

Domain 2: Risk of bias due to deviations from the intended interventions (*effect of assignment to intervention*)

| **Signalling questions** | **Comments** | **Response options** |
| --- | --- | --- |
| **2.1. Were participants aware of their assigned intervention during the trial?** | Treating physician unblinded; observer-blinded study design only applied to outcome assessor. | Y |
| **2.2. Were carers and people delivering the interventions aware of participants' assigned intervention during the trial?** |  | Y |
| **2.3. If Y/PY/NI to 2.1 or 2.2: Were there deviations from the intended intervention that arose because of the trial context?** | No deviations from intended intervention reported; protocol followed as planned. | N |
| **2.4 If Y/PY to 2.3: Were these deviations likely to have affected the outcome?** |  | NA |
| **2.5. If Y/PY/NI to 2.4: Were these deviations from intended intervention balanced between groups?** |  | NA |
| **2.6 Was an appropriate analysis used to estimate the effect of assignment to intervention?** | Paired analyses (t-tests, ANOVA) comparing laser-treated vs. control regions within the same patient were used, appropriate for the split-face design. | Y |
| **2.7 If N/PN/NI to 2.6: Was there potential for a substantial impact (on the result) of the failure to analyse participants in the group to which they were randomized?** |  | NA |
| **Risk-of-bias judgement** | Unblinded participants and carers may introduce performance bias, but split-face design and blinded assessor partially mitigate this; no protocol deviations. | Some concerns |
| Optional: What is the predicted direction of bias due to deviations from intended interventions? |  | NA |

Domain 2: Risk of bias due to deviations from the intended interventions (*effect of adhering to intervention*)

| **Signalling questions** | **Comments** | **Response options** |
| --- | --- | --- |
| **2.1. Were participants aware of their assigned intervention during the trial?** | Participants were not blinded; observer-blinded design only applied to outcome assessor.  Treating physician unblinded; observer-blinded design only applied to outcome assessor. | Y |
| **2.2. Were carers and people delivering the interventions aware of participants' assigned intervention during the trial?** |  | Y |
| **2.3. [If applicable:] If Y/PY/NI to 2.1 or 2.2: Were important non-protocol interventions balanced across intervention groups?** | Split-face design ensures co-interventions (sunscreen, moisturizer) were applied equally to both sides. | Y |
| **2.4. [If applicable:] Were there failures in implementing the intervention that could have affected the outcome?** | Intervention delivered as per protocol; no failures in implementation reported. | N |
| **2.5. [If applicable:] Was there non-adherence to the assigned intervention regimen that could have affected participants’ outcomes?** | Non-adherence not reported. | N |
| **2.6. If N/PN/NI to 2.3, or Y/PY/NI to 2.4 or 2.5: Was an appropriate analysis used to estimate the effect of adhering to the intervention?** |  | NA |
| **Risk-of-bias judgement** |  | Some concerns |
| Optional: What is the predicted direction of bias due to deviations from intended interventions? | Unblinded participants and carers may have introduced performance bias favouring the laser intervention. | Away from null |

Domain 3: Missing outcome data

| **Signalling questions** | **Comments** | **Response options** |
| --- | --- | --- |
| **3.1 Were data for this outcome available for all, or nearly all, participants randomized?** | One dropout due to pain (1/14). Primary outcome data available for all remaining participants. Missingness unlikely to be related to the outcome. | Y |
| **3.2 If N/PN/NI to 3.1: Is there evidence that the result was not biased by missing outcome data?** |  | NA |
| **3.3 If N/PN to 3.2: Could missingness in the outcome depend on its true value?** |  | NA |
| **3.4 If Y/PY/NI to 3.3: Is it likely that missingness in the outcome depended on its true value?** |  | NA |
| **Risk-of-bias judgement** |  | Low |
| Optional: What is the predicted direction of bias due to missing outcome data? |  | NA |

Domain 4: Risk of bias in measurement of the outcome

| **Signalling questions** | **Comments** | **Response options** |
| --- | --- | --- |
| **4.1 Was the method of measuring the outcome inappropriate?** | Multiple appropriate outcome measures used: blinded physician assessment (PhGA), reflectance spectroscopy, melanin index, and blinded histopathology. Methods are well-established for assessing pigmentation changes. | N |
| **4.2 Could measurement or ascertainment of the outcome have differed between intervention groups?** | Blinded outcome assessment (physician and pathologist) and objective measurements (spectroscopy, melanin index) used; measurement methods did not differ between groups. | N |
| **4.3 If N/PN/NI to 4.1 and 4.2: Were outcome assessors aware of the intervention received by study participants?** | Physician and pathologist outcome assessors were blinded to treatment allocation. | N |
| **4.4 If Y/PY/NI to 4.3: Could assessment of the outcome have been influenced by knowledge of intervention received?** |  | NA |
| **4.5 If Y/PY/NI to 4.4:** **Is it likely that assessment of the outcome was influenced by knowledge of intervention received?** |  | NA |
| **Risk-of-bias judgement** |  | Low |
| Optional: What is the predicted direction of bias in measurement of the outcome? |  | NA |

Domain 5: Risk of bias in selection of the reported result

| **Signalling questions** | **Comments** | **Response options** |
| --- | --- | --- |
| **5.1 Were the data that produced this result analysed in accordance with a pre-specified analysis plan that was finalized before unblinded outcome data were available for analysis?** | No mention of a pre-specified analysis plan or protocol registration. | NI |
| **Is the numerical result being assessed likely to have been selected, on the basis of the results, from...** |  |  |
| **5.2. ... multiple eligible outcome measurements (e.g. scales, definitions, time points) within the outcome domain?** | Multiple outcome measures were assessed (PhGA, melanin index, spectroscopy, histopathology, patient outcomes). However, the primary outcome (PhGA) was pre-specified and all outcomes were reported; selective reporting is unlikely. | N |
| **5.3 ... multiple eligible analyses of the data?** | Single pre-specified analysis plan appears to have been used; no indication of multiple analyses or selective reporting. | N |
| **Risk-of-bias judgement** |  | Low |
| Optional: What is the predicted direction of bias due to selection of the reported result? |  | NA |

Overall risk of bias

| **Risk-of-bias judgement** |  | Some concerns |
| --- | --- | --- |
| Optional: What is the overall predicted direction of bias for this outcome? |  | NA |

This work is licensed under a [Creative Commons Attribution-NonCommercial-NoDerivatives 4.0 International License](http://creativecommons.org/licenses/by-nc-nd/4.0/)

Revised Cochrane risk-of-bias tool for randomized trials (RoB 2)

TEMPLATE FOR COMPLETION

Edited by Julian PT Higgins, Jelena Savović, Matthew J Page, Jonathan AC Sterne
on behalf of the RoB2 Development Group

**Version of 22 August 2019**

Reviewers: Virgilio Blandon; Halyma Gomez

The development of the RoB 2 tool was supported by the MRC Network of Hubs for Trials Methodology Research (MR/L004933/2- N61), with the support of the host MRC ConDuCT-II Hub (Collaboration and innovation for Difficult and Complex randomised controlled Trials In Invasive procedures - MR/K025643/1), by MRC research grant MR/M025209/1, and by a grant from The Cochrane Collaboration.

This work is licensed under a [Creative Commons Attribution-NonCommercial-NoDerivatives 4.0 International License](http://creativecommons.org/licenses/by-nc-nd/4.0/).

**Study details**

| **Reference** | Wind BS, Meesters AA, Kroon MW, Beek JF, van der Veen JP, van der Wal AC, Bos JD, Wolkerstorfer A. Formation of fibrosis after nonablative and ablative fractional laser therapy. Dermatol Surg. 2012 Mar;38(3):437-42. doi: 10.1111/j.1524-4725.2011.02230.x. Epub 2011 Nov 28. PMID: 22122738. |
| --- | --- |

**Study design**

| □ | Individually-randomized parallel-group trial |
| --- | --- |
| □ | Cluster-randomized parallel-group trial |
| X | Individually randomized cross-over (or other matched) trial |

**For the purposes of this assessment, the interventions being compared are defined as**

| Experimental: | Patients in the experimental group received active fractional laser therapy combined with intermittent topical bleaching. For patients with ashy dermatosis (AD) and postinflammatory hyperpigmentation (PIH), treatment consisted of four to five sessions of nonablative 1550 nm fractional laser therapy using the Fraxel re:store laser, with a pulse energy of 15 mJ per microbeam, coverage ranging from 14% to 20% depending on Fitzpatrick skin type, and eight passes per session. For patients with Becker's nevus (BN), the experimental intervention was three sessions of ablative 10600 nm fractional laser therapy using the Fraxel re:pair laser, with a pulse energy of 10 mJ per microbeam, coverage of 35% to 45%, and four passes per session. In all patients, the laser-treated region also received the same intermittent regimen of topical bleaching cream containing hydroquinone 5%, tretinoin 0.05%, and triamcinolone acetonide 0.1%, applied once daily in the evening during the second and third weeks after each laser treatment. The experimental regions were randomized to one side of each patient's affected area, and biopsies were obtained from the optical center of the treated site three months after the last laser treatment to assess for dermal fibrosis. | Comparator: | Patients in the comparator group received topical bleaching alone without any laser therapy. The same intermittent regimen of triple combination topical cream (hydroquinone 5%, tretinoin 0.05%, and triamcinolone acetonide 0.1%) was applied once daily in the evening to the control region during the second and third weeks after each laser treatment session. These control regions were located on the contralateral side of each patient's affected area and were randomized to receive no laser exposure. Biopsies were obtained from the optical center of the control site three months after the last laser treatment to allow comparison of histologic outcomes, particularly the presence or absence of dermal fibrosis, with the laser-treated regions. The split-face design ensured that co-interventions such as sunscreen application, moisturizer use, and other skincare measures were applied equally to both the experimental and comparator regions. |
| --- | --- | --- | --- |

| **Specify which outcome is being assessed for risk of bias** | The outcome being assessed for risk of bias is the formation of dermal fibrosis, evaluated through histopathological examination of skin biopsies obtained three months after the last laser treatment. The extent of fibrosis was determined by a blinded dermatopathologist using hematoxylin and eosin staining, with fibrosis scored on a three-point scale where 0 indicated absence, 1 indicated minimal presence, and 2 indicated definite presence of fibrosis. The numerical result being assessed is the comparison of fibrosis occurrence between treatment groups, with fibrosis observed in four of eight patients (50%) treated with ablative fractional laser therapy and in zero of ten patients (0%) treated with nonablative fractional laser therapy, a difference that was statistically significant (p < 0.05), as reported in the results section under the headings "Nonablative Fractional Laser" and "Ablative Fractional Laser" (Wind et al., 2012, pp. 439-440). |
| --- | --- |

| **Specify the numerical result being assessed.** In case of multiple alternative analyses being presented, specify the numeric result (e.g. RR = 1.52 (95% CI 0.83 to 2.77) and/or a reference (e.g. to a table, figure or paragraph) that uniquely defines the result being assessed. | The numerical result being assessed is the comparison of dermal fibrosis occurrence between treatment groups. Dermal fibrosis was observed in four of eight patients (50%) treated with ablative fractional laser therapy and in zero of ten patients (0%) treated with nonablative fractional laser therapy, a difference that was statistically significant (p < 0.05), as reported in the results section under the headings "Nonablative Fractional Laser" and "Ablative Fractional Laser" (Wind et al., 2012, p. 440). No effect estimate such as relative risk or confidence interval is reported; only the proportions and the p-value are provided. |
| --- | --- |

**Is the review team’s aim for this result…?**

| X | to assess the effect of *assignment to intervention* (the ‘intention-to-treat’ effect) |
| --- | --- |
| □ | to assess the effect of *adhering to intervention* (the ‘per-protocol’ effect) |

**If the aim is to assess the effect of *adhering to intervention***, select the deviations from intended intervention that should be addressed (at least one must be checked):

□	occurrence of non-protocol interventions

□	failures in implementing the intervention that could have affected the outcome

□	non-adherence to their assigned intervention by trial participants

**Which of the following sources were obtained to help inform the risk-of-bias assessment? (tick as many as apply)**

X	Journal article(s) with results of the trial

□	Trial protocol

□	Statistical analysis plan (SAP)

□	Non-commercial trial registry record (e.g. ClinicalTrials.gov record)

□	Company-owned trial registry record (e.g. GSK Clinical Study Register record)

□	“Grey literature” (e.g. unpublished thesis)

□	Conference abstract(s) about the trial

□	Regulatory document (e.g. Clinical Study Report, Drug Approval Package)

□	Research ethics application

□	Grant database summary (e.g. NIH RePORTER or Research Councils UK Gateway to Research)

□	Personal communication with trialist

□	Personal communication with the sponsor

## Risk of bias assessment

Responses underlined in green are potential markers for low risk of bias, and responses in red are potential markers for a risk of bias. Where questions relate only to sign posts to other questions, no formatting is used.

**Domain 1: Risk of bias arising from the randomization process**

| **Signalling questions** | **Comments** | **Response options** |
| --- | --- | --- |
| **1.1 Was the allocation sequence random?** | Randomization used digitally created random list and sealed envelopes opened in ascending order.  Sealed envelopes opened in ascending order with digitally created random list; likely concealed but not explicitly described as opaque or sequentially numbered. | Y |
| **1.2 Was the allocation sequence concealed until participants were enrolled and assigned to interventions?** |  | PY |
| **1.3 Did baseline differences between intervention groups suggest a problem with the randomization process?** | Split-face design ensures inherent balance; same patient serves as both experimental and control, and regions were described as "similar." | N |
| **Risk-of-bias judgement** |  | Low |
| Optional: What is the predicted direction of bias arising from the randomization process? |  | NA |

Domain 2: Risk of bias due to deviations from the intended interventions (*effect of assignment to intervention*)

| **Signalling questions** | **Comments** | **Response options** |
| --- | --- | --- |
| **2.1. Were participants aware of their assigned intervention during the trial?** | Participants were not blinded; observer-blinded design only applied to outcome assessor.  Treating physician unblinded; observer-blinded design only applied to outcome assessor. | Y |
| **2.2. Were carers and people delivering the interventions aware of participants' assigned intervention during the trial?** |  | Y |
| **2.3. If Y/PY/NI to 2.1 or 2.2: Were there deviations from the intended intervention that arose because of the trial context?** | No deviations from intended intervention reported; protocol followed as planned. | N |
| **2.4 If Y/PY to 2.3: Were these deviations likely to have affected the outcome?** |  | NA |
| **2.5. If Y/PY/NI to 2.4: Were these deviations from intended intervention balanced between groups?** |  | NA |
| **2.6 Was an appropriate analysis used to estimate the effect of assignment to intervention?** | Paired analysis (t-test) used to compare fibrosis occurrence between treated and control regions within the same patient, appropriate for split-face design. | Y |
| **2.7 If N/PN/NI to 2.6: Was there potential for a substantial impact (on the result) of the failure to analyse participants in the group to which they were randomized?** |  | NA |
| **Risk-of-bias judgement** |  | Some concerns |
| Optional: What is the predicted direction of bias due to deviations from intended interventions? |  | NA |

Domain 2: Risk of bias due to deviations from the intended interventions (*effect of adhering to intervention*)

| **Signalling questions** | **Comments** | **Response options** |
| --- | --- | --- |
| **2.1. Were participants aware of their assigned intervention during the trial?** | Participants were not blinded; observer-blinded design only applied to outcome assessor.  Treating physician unblinded; observer-blinded design only applied to outcome assessor. | Y |
| **2.2. Were carers and people delivering the interventions aware of participants' assigned intervention during the trial?** |  | Y |
| **2.3. [If applicable:] If Y/PY/NI to 2.1 or 2.2: Were important non-protocol interventions balanced across intervention groups?** | Split-face design ensures co-interventions (sunscreen, moisturizer) were applied equally to both sides. | Y |
| **2.4. [If applicable:] Were there failures in implementing the intervention that could have affected the outcome?** | Intervention delivered as per protocol; no failures in implementation reported. | N |
| **2.5. [If applicable:] Was there non-adherence to the assigned intervention regimen that could have affected participants’ outcomes?** | Non-adherence not reported. | N |
| **2.6. If N/PN/NI to 2.3, or Y/PY/NI to 2.4 or 2.5: Was an appropriate analysis used to estimate the effect of adhering to the intervention?** |  | NA |
| **Risk-of-bias judgement** |  | Some concerns |
| Optional: What is the predicted direction of bias due to deviations from intended interventions? |  | NA |

Domain 3: Missing outcome data

| **Signalling questions** | **Comments** | **Response options** |
| --- | --- | --- |
| **3.1 Were data for this outcome available for all, or nearly all, participants randomized?** | Missing data for 7 of 25 patients (28%). Reason for missingness not explained. Selection of biopsied patients may be related to factors associated with fibrosis outcome. | PN |
| **3.2 If N/PN/NI to 3.1: Is there evidence that the result was not biased by missing outcome data?** | No sensitivity analysis performed; missingness mechanism unknown; reason for missing biopsy data (7/25 patients) not explained and may be related to fibrosis outcome. | N |
| **3.3 If N/PN to 3.2: Could missingness in the outcome depend on its true value?** | No evidence that missing biopsy data were related to the fibrosis outcome; likely due to study design (substudy selection) rather than outcome values. | N |
| **3.4 If Y/PY/NI to 3.3: Is it likely that missingness in the outcome depended on its true value?** |  | NA |
| **Risk-of-bias judgement** |  | High |
| Optional: What is the predicted direction of bias due to missing outcome data? |  | NA |

Domain 4: Risk of bias in measurement of the outcome

| **Signalling questions** | **Comments** | **Response options** |
| --- | --- | --- |
| **4.1 Was the method of measuring the outcome inappropriate?** | Histological evaluation (H&E) by a blinded dermatopathologist using a standardized scoring system is an appropriate method for assessing dermal fibrosis. | N |
| **4.2 Could measurement or ascertainment of the outcome have differed between intervention groups?** | Blinded histopathological assessment used for both groups; measurement methods did not differ between intervention groups. | N |
| **4.3 If N/PN/NI to 4.1 and 4.2: Were outcome assessors aware of the intervention received by study participants?** | Outcome assessor (dermatopathologist) was blinded to treatment allocation. | N |
| **4.4 If Y/PY/NI to 4.3: Could assessment of the outcome have been influenced by knowledge of intervention received?** |  | NA |
| **4.5 If Y/PY/NI to 4.4: Is it likely that assessment of the outcome was influenced by knowledge of intervention received?** |  | NA |
| **Risk-of-bias judgement** |  | Low |
| Optional: What is the predicted direction of bias in measurement of the outcome? |  | NA |

Domain 5: Risk of bias in selection of the reported result

| **Signalling questions** | **Comments** | **Response options** |
| --- | --- | --- |
| **5.1 Were the data that produced this result analysed in accordance with a pre-specified analysis plan that was finalized before unblinded outcome data were available for analysis?** | No mention of a pre-specified analysis plan or protocol registration. | NI |
| **Is the numerical result being assessed likely to have been selected, on the basis of the results, from...** |  |  |
| **5.2. ... multiple eligible outcome measurements (e.g. scales, definitions, time points) within the outcome domain?** | Single outcome measurement (fibrosis) with one definition and time point. | N |
| **5.3 ... multiple eligible analyses of the data?** | Single pre-specified analysis plan appears to have been used; no indication of multiple analyses or selective reporting. | N |
| **Risk-of-bias judgement** |  | Low |
| Optional: What is the predicted direction of bias due to selection of the reported result? |  | NA |

Overall risk of bias

| **Risk-of-bias judgement** | High risk of bias due to substantial missing outcome data (7/25 patients, 28%) without explanation or sensitivity analysis. Additionally, the comparison between nonablative and ablative lasers was not randomized; the two groups came from different patient populations (AD/PIH vs. BN). | High |
| --- | --- | --- |
| Optional: What is the overall predicted direction of bias for this outcome? |  | NA |

This work is licensed under a [Creative Commons Attribution-NonCommercial-NoDerivatives 4.0 International License](http://creativecommons.org/licenses/by-nc-nd/4.0/)
