# Supplementary material for: Non-ablative dermal remodeling lasers in acquired dermal macular hyperpigmentation: A scoping review
Source: PLoS One. 2026 Sep 28;21(9):e0359552. doi: 10.1371/journal.pone.0359552 (PMC13618957; doi:10.1371/journal.pone.0359552)
Supplement: Supplementary file 3 [file pone.0359552.s004.docx]

# **S1 Table. Search Strategy and Results Across All Information Sources (Electronic Databases and Citation Tracking)**

| **Dataset** | **Date** | **Search-string** | **Results** |  |
| --- | --- | --- | --- | --- |
| Pubmed | 2/6/2026 | ("Hyperpigmentation"[Mesh] OR "erythema dyschromicum perstans"[tiab] OR "ashy dermatosis"[tiab] OR "dermatosis cenicienta"[tiab] OR "acquired dermal macular hyperpigmentation"[tiab]) AND ("Lasers, Solid-State"[Mesh] OR "Nd:YAG"[tiab] OR "neodymium-yag"[tiab] OR "laser therapy"[tiab] OR "laser treatment"[tiab] OR "laser"[tiab]) AND ("1320 nm"[tiab] OR "1320-nm"[tiab] OR "1320nm"[tiab]) | 0 |  |
| Pubmed | 2/6/2026 | (Acquired Dermal Macular Hyperpigmentation[Mesh] OR "Erythema Dyschromicum Perstans"[tiab] OR "Lichen Planus Pigmentosus"[tiab] OR ashy dermatosis[tiab] OR dermatosis cenicienta[tiab] OR EDP[tiab] OR LPP[tiab] OR "idiopathic eruptive macular pigmentation"[tiab] OR "pigmented contact dermatitis"[tiab] OR "Riehl's melanosis"[tiab] OR "acquired dermal macular hyperpigmentation"[tiab]) AND (Lasers[Mesh] OR "Lasers, Solid-State"[Mesh] OR "laser therapy"[tiab] OR "laser treatment"[tiab] OR "Nd:YAG"[tiab] OR "neodymium-yag"[tiab] OR "Q-switched"[tiab] OR picosecond[tiab] OR "fractional laser"[tiab] OR "non-ablative laser"[tiab] OR laser[tiab]) | 131 |  |
| Pubmed | 2/6/2026 | ( "Acquired Dermal Macular Hyperpigmentation"[tiab] OR "erythema dyschromicum perstans"[tiab] OR "ashy dermatosis"[tiab] OR "dermatosis cenicienta"[tiab] OR "lichen planus pigmentosus"[tiab] OR "Riehl's melanosis"[tiab] OR "pigmented contact dermatitis"[tiab] ) AND ( "laser therapy"[tiab] OR "laser treatment"[tiab] OR laser[tiab] ) AND ( "1320 nm"[tiab] OR "1320-nm"[tiab] OR "1450 nm"[tiab] OR "1450-nm"[tiab] OR "1540 nm"[tiab] OR "1550 nm"[tiab] OR "1550-nm"[tiab] OR "1565 nm"[tiab] OR "1570 nm"[tiab] OR "non-ablative"[tiab] OR "nonablative"[tiab] OR "fractional laser"[tiab] OR "dermal remodeling"[tiab] OR "water-targeting"[tiab] OR "1470nm"[tiab] OR "980nm"[tiab] OR "1470-nm"[tiab] OR "980-nm"[tiab] OR "1410nm"[tiab] OR "1410-nm"[tiab] OR "1410-nm"[tiab] OR "1540nm"[tiab] OR "1540-nm"[tiab] OR "1927nm"[tiab] OR "1927-nm"[tiab] OR "1940nm"[tiab] OR "1940-nm"[tiab] OR "1444nm"[tiab] OR "1444-nm"[tiab] ) | 7 |  |
| Scielo | 2/6/2026 | dermatosis cenicienta laser | 0 |  |
| Scielo | 2/6/2026 | eritema discrómico perstans laser | 0 |  |
| Scielo | 2/6/2026 | liquen plano pigmentoso laser | 0 |  |
| Scielo | 2/6/2026 | eritema dischromico perstans laser | 0 |  |
| Scielo | 2/6/2026 | liquen plano pigmentoso laser | 0 |  |
| Scielo | 2/6/2026 | Ashy dermatosis laser | 0 |  |
| Scielo | 2/6/2026 | lichen planus pigmentosus laser | 0 |  |
| Scielo | 2/6/2026 | dermatosis cenicienta laser Nd:YAG | 0 |  |
| Scielo | 2/6/2026 | laser pigmentacion piel | 0 |  |
| Scielo | 2/6/2026 | Nd:YAG pigmentacion | 0 |  |
| Scielo | 2/6/2026 | Nd:YAG hiperpigmentacion | 0 |  |
| LILACS | 2/6/2026 | ("dermatosis cenicienta" OR "eritema discrómico perstans") AND ("Nd:YAG" OR "laser Nd:YAG") | 0 |  |
| LILACS | 2/6/2026 | ("liquen plano pigmentoso" OR "melanosis de Riehl") AND ("Nd:YAG" OR laser) | 1 |  |
| LILACS | 2/6/2026 | ("Nd:YAG" AND 1320) | 1 |  |
| LILACS | 2/6/2026 | ("laser 1320 nm") | 0 |  |
| LILACS | 2/6/2026 | hiperpigmentacion Nd:YAG | 0 |  |
| LILACS | 2/6/2026 | pigmentacion laser Nd:YAG | 0 |  |
| LILACS | 2/6/2026 | ("ashy dermatosis" OR "erythema dyschromicum perstans") AND ("Nd:YAG") | 0 |  |
| LILACS | 2/6/2026 | "lichen planus pigmentosus" AND laser | 0 |  |
| LILACS | 2/6/2026 | dermatosis cenicienta Nd:YAG | 0 |  |
| LILACS | 2/6/2026 | lichen planus pigmentosus laser | 0 |  |
| LILACS | 2/6/2026 | Riehl melanosis laser | 1 |  |
| DOAJ | 2/6/2026 | ("ashy dermatosis" OR "erythema dyschromicum perstans" OR "lichen planus pigmentosus") AND ("Nd:YAG" OR laser) | 0 |  |
| DOAJ | 2/6/2026 | ("Riehl's melanosis" OR "idiopathic eruptive macular pigmentation") AND ("Nd:YAG" OR laser) | 0 |  |
| DOAJ | 2/6/2026 | ashy dermatosis erythema dyschromicum perstans lichen planus pigmentosus laser Nd:YAG | 0 |  |
| DOAJ | 2/6/2026 | Riehl's melanosis idiopathic eruptive macular pigmentation laser Nd:YAG | 0 |  |
| DOAJ | 2/6/2026 | ("Nd:YAG" AND "1320 nm") | 0 |  |
| DOAJ | 2/6/2026 | "1320 nm laser pigmentation" | 0 |  |
| DOAJ | 2/6/2026 | "Riehl's melanosis" laser | 2 |  |
| DOAJ | 2/6/2026 | "idiopathic eruptive macular pigmentation" laser | 0 |  |
| DOAJ | 2/6/2026 | "lichen planus pigmentosus" laser | 7 |  |
| DOAJ | 2/6/2026 | "ashy dermatosis" laser | 1 |  |
| ClinicalTrials.gov | 2/6/2026 | Condition/disease: erythema dyschromicum perstans | 1 |  |
| ClinicalTrials.gov | 2/6/2026 | Condition/disease: ashy dermatosis | 1 |  |
| ClinicalTrials.gov | 2/6/2026 | Condition/disease: lichen planus pigmentosus | 3 |  |
| ClinicalTrials.gov | 2/6/2026 | Condition/disease: idiopathic eruptive macular pigmentation | 0 |  |
| ClinicalTrials.gov | 2/6/2026 | Condition/disease: Riehl’s melanosis | 0 |  |
| Clinicaltrials.eu | 2/6/2026 | erythema dyschromicum perstans | 0 |  |
| Clinicaltrials.eu | 2/6/2026 | ashy dermatosis | 0 |  |
| Clinicaltrials.eu | 2/6/2026 | lichen planus pigmentosus | 0 |  |
| Clinicaltrials.eu | 2/6/2026 | Riehl’s melanosis | 0 |  |
| Clinicaltrials.eu | 2/6/2026 | idiopathic eruptive macular pigmentation | 0 |  |
| WHO ICTRP | 2/6/2026 | erythema dyschromicum perstans | 1 |  |
| WHO ICTRP | 2/6/2026 | ashy dermatosis | 2 |  |
| WHO ICTRP | 2/6/2026 | lichen planus pigmentosus | 18 |  |
| WHO ICTRP | 2/6/2026 | Riehl’s melanosis | 1 |  |
| WHO ICTRP | 2/6/2026 | idiopathic eruptive macular pigmentation | 0 |  |
| Cochrane central | 2/6/2026 | lichen planus pigmentosus laser | 7 |  |
| Cochrane central | 2/6/2026 | idiopathic eruptive macular pigmentation laser | 0 |  |
| Cochrane central | 2/6/2026 | Riehl’s melanosis laser | 2 |  |
| Cochrane central | 2/6/2026 | ashy dermatosis laser | 4 |  |
| Cochrane central | 2/6/2026 | erythema dyschromicum perstans laser | 2 |  |
| Subtotal –Electronic Databases |  |  | 193 |  |
| Citation Tracking | N/A | Backward | 914 |  |
| Citation Tracking | 22/06/2026 | Forward | 160 |  |
| Subtotal –Citation Tracking |  |  | 1074 |  |
| **Total** | **1267** | | |  |

Note: The search was conducted on June 2, 2026, across PubMed, SciELO, LILACS, DOAJ, Cochrane central, ClinicalTrials.gov, ClinicalTrials.eu, and the WHO ICTRP. Search strings combined a broad set of terms for pigmentary disorders (e.g., ashy dermatosis, erythema dyschromicum perstans, lichen planus pigmentosus, Riehl's melanosis, and acquired dermal macular hyperpigmentation) with terms for laser therapies (covering various modalities and wavelengths), adapted to the language and syntax of each database. For citation tracking: backward searching was performed on all review articles identified from the primary database searches, without exception. Additionally, both backward and forward searching were performed on all articles that were ultimately included after the title/abstract and full-text screening process. The total number of records retrieved from all sources was 1,267.
