# Supplementary material for: Non-ablative dermal remodeling lasers in acquired dermal macular hyperpigmentation: A scoping review
Source: PLoS One. 2026 Sep 28;21(9):e0359552. doi: 10.1371/journal.pone.0359552 (PMC13618957; doi:10.1371/journal.pone.0359552)
Supplement: Supplementary file 4 [file pone.0359552.s005.docx]

# **S2 Table. Summary of article inclusion, deduplication, and exclusion during the selection process.**

| **Category** | **Count** | **Notes** |
| --- | --- | --- |
| Total records retrieved (before deduplication) | 193 | Sum of all database search results prior to deduplication |
| Duplicates removed | 33 | Identified during deduplication |
| Unique records after deduplication | 160 | Total records screened at title/abstract level |
| Screening exclusions – Wrong condition (e.g. melasma, lentigos, post‑inflammatory hyperpigmentation) | 15 | E1 |
| Screening exclusions – Inadequate intervention (e.g., alexandrite, CO₂, IPL). | 20 | E2 |
| Screening exclusions – Full text not retrievable | 0 | E3 |
| Screening exclusions – Duplicate data (secondary publication of the same clinical data) | 4 | E4 |
| Screening exclusions – Preclinical study or secondary research (without primary data) | 86 | E5 |
| Screening exclusions – Wrong language (not English, Spanish, or Portuguese) | 1 | E6 |
| Screening exclusions – No original patient data (e.g., editorial, commentary, letter without extractable data) | 1 | E7 |
| Subtotal of excluded after screening | 127 | Sum of E1–E7 |
| Included for full-text review | 33 | I |

This table summarizes the full selection process for studies included in the review. It reports the total number of records retrieved before deduplication, the number of duplicate and unique records identified, and the distribution of exclusions at the title and abstract screening stage. Exclusion reasons are provided according to predefined categories, including population outside the predefined conditions, review/editorial articles, conference abstracts, animal studies, inadequate interventions, insufficient data, lack of full-text availability, and inappropriate study design. Articles meeting eligibility criteria at this stage were included for full-text review.
