# Supplementary material for: Non-ablative dermal remodeling lasers in acquired dermal macular hyperpigmentation: A scoping review
Source: PLoS One. 2026 Sep 28;21(9):e0359552. doi: 10.1371/journal.pone.0359552 (PMC13618957; doi:10.1371/journal.pone.0359552)
Supplement: Supplementary file 5 [file pone.0359552.s006.docx]

# **S3 Table. Full-text review inclusion and exclusion**

| **Reason for Exclusion / Status** | **Number of Articles** |
| --- | --- |
| Wrong condition | 0 |
| Wrong intervention | 24 |
| Full text not retrievable | 0 |
| Duplicate data | 0 |
| Preclinical study or secondary research | 0 |
| Wrong language | 0 |
| No original patient data | 2 |
| **Subtotal of Excluded** | 26 |
| **Included for final review** | 7 |
| **Total full-text articles assessed** | 33 |

This table summarizes the outcomes of the full-text screening phase. It reports the number of articles excluded after full-text assessment along with the reasons for exclusion, based on predefined eligibility criteria. The table provides a quantitative overview of inclusion and exclusion decisions at the full-text screening stage.
