# Supplementary material for: Non-ablative dermal remodeling lasers in acquired dermal macular hyperpigmentation: A scoping review
Source: PLoS One. 2026 Sep 28;21(9):e0359552. doi: 10.1371/journal.pone.0359552 (PMC13618957; doi:10.1371/journal.pone.0359552)
Supplement: Supplementary file 6 [file pone.0359552.s007.docx]

# **S4 Table. Summary of backward and forward citation searches across databases**

| **Round** | **Date of search (forward only)** | **Source Article (ID)** | **First author** | **Search Type** | **Total results** | **Meet Inclusion** |
| --- | --- | --- | --- | --- | --- | --- |
| 1 | N/A | Pubmed-1 | Sathe NCBen Tanfous A | Backward | 61 | 0 |
| 2 | N/A | Pubmed-2 | Xu L | Backward | 47 | 0 |
| 3 | N/A | Pubmed-3 | Ding Y | Backward | 75 | 0 |
| 4 | N/A | Pubmed-4 | Aurangabadkar SJ | Backward | 24 | 0 |
| 5 | N/A | Pubmed-7 | Zhang AD | Backward | 99 | 0 |
| 6 | N/A | Pubmed-9 | Pérez-Bernal A | Backward | 31 | 0 |
| 7 | N/A | Pubmed-10 | Lautenschlager S | Backward | 63 | 0 |
| 8 | N/A | Pubmed-13 | Ezekwe U | Backward | 22 | 0 |
| 9 | N/A | Pubmed-16 | Garg S | Backward | 65 | 0 |
| 10 | N/A | Pubmed-22 | Tanha AE | Backward | 59 | 0 |
| 11 | N/A | Pubmed-24 | Stratigos AJ | Backward | 49 | 0 |
| 12 | N/A | Pubmed-27 | Farabi B | Backward | 36 | 0 |
| 13 | N/A | Pubmed-42 | Abdel-Naser MB | Backward | 59 | 0 |
| 14 | N/A | Pubmed-55 | Sarkar R | Backward | 54 | 0 |
| 15 | N/A | Pubmed-56 | Wolf-de Jonge IC | Backward | 87 | 0 |
| 16 | N/A | Pubmed-8 | Kim SM | Backward | 19 | 0 |
| 17 | N/A | Pubmed-11 | Wolfshohl JA | Backward | 18 | 0 |
| 18 | N/A | Pubmed-26 | Kroon MW | Backward | 20 | 0 |
| 19 | N/A | Pubmed-30 | Wind BS | Backward | 11 | 0 |
| 20 | N/A | Pubmed-49 | Jung CJ | Backward | 10 | 0 |
| 21 | N/A | Pubmed-62 | Murray TN | Backward | 5 | 0 |
| 22 | June 22th of 2026 | Pubmed-63 | Kim SM | Forward | 22 | 0 |
| 23 | June 22th of 2026 | Pubmed-64 | Wolfshohl JA | Forward | 29 | 0 |
| 24 | June 22th of 2026 | Pubmed-65 | Garg S | Forward | 34 | 0 |
| 25 | June 22th of 2026 | Pubmed-66 | Kroon MW | Forward | 40 | 0 |
| 26 | June 22th of 2026 | Pubmed-67 | Wind BS | Forward | 28 | 0 |
| 27 | June 22th of 2026 | Pubmed-68 | Jung CJ | Forward | 6 | 0 |
| 28 | June 22th of 2026 | Pubmed-69 | Murray TN | Forward | 1 | 0 |

Note. This table reports the results of backward and forward citation searches performed on selected source articles to identify additional studies potentially eligible for inclusion. For each round, the table provides the source article ID, date of the search (forward searches only), search type (backward or forward), total results retrieved and number of articles meeting inclusion criteria.
