# Supplementary material for: Non-ablative dermal remodeling lasers in acquired dermal macular hyperpigmentation: A scoping review
Source: PLoS One. 2026 Sep 28;21(9):e0359552. doi: 10.1371/journal.pone.0359552 (PMC13618957; doi:10.1371/journal.pone.0359552)
Supplement: Supplementary file 7 [file pone.0359552.s008.docx]

**S5 Table.** **Comprehensive study details of included articles on laser therapy for acquired dermal macular hyperpigmentation (ADMH)**

| **Author, Year** | **Condition** | **Laser Device & Wavelength** | **Treatment Parameters** | **Adjunctive Therapies** | **Study Design** | **Sample Size** | **Clinical Outcomes** | **Histological Findings (if reported)** | **Patient-Reported Outcomes** | **Adverse Events** | **Limitations** | **Key Conclusions** | **Sources of Funding** |
| --- | --- | --- | --- | --- | --- | --- | --- | --- | --- | --- | --- | --- | --- |
| Kroon MW, et al., 2012 | EDP and PIH | 1550 nm non-ablative (Fraxel re:store). | 1550 nm non-ablative (Fraxel re:store); 15 mJ/MTZ; 8 passes; 14–20% coverage; 5 sessions at 3-week intervals; topical anesthesia; cooling. | Intermittent topical bleaching: hydroquinone 5%, tretinoin 0.05%, triamcinolone 0.1% applied once daily during weeks 2–3 post-laser. | Randomized controlled observer-blinded study with split-lesion design | 8/14 patients (8 EDP*, 6 PIH); 1 dropout (EDP, after 1 session due to pain); 13 completed | No clinical improvement observed. PhGA showed no significant difference between laser-treated and control sites. Reflectance spectroscopy (L-value) and melanin index showed no significant differences. No recurrence data reported. | 3-month biopsy: No significant differences in melanocyte count (Melan A) or dermal melanin (Fontana) between laser-treated and control sites. | Patient-reported outcomes reported. PhGA (VAS 0–10): EDP 3.6; PIH 4.5. Satisfaction (VAS 0–10): EDP 5.7; PIH 4.5. Pain (VAS 0–10): 5.4. Would recommend: EDP 2/7; PIH 4/6. | Direct side effects: erythema (72%), burning sensation (73%), edema (7%), crusting (2%). Laser-induced PIH developed in 3 patients (23%): 1 EDP (flank) and 2 PIH (forearm, chest) after 2–3 sessions; persistent at 3-month follow-up. | Small sample; short follow-up; heterogeneous population (EDP/PIH); no dose-finding; cooling may affect efficacy; seasonal treatment may increase PIH risk; histopathology not available for all. | 1550 nm FLT not effective for EDP/PIH; 23% PIH. Not recommended. | Laser equipment and disposables provided by B&Co Laser Medico and Solta Medical Inc. exclusively for research purposes; no role in study design, conduct, analysis, or manuscript preparation. No other funding sources declared. Authors report no conflicts of interest. |
| Wind BS, et al., 2012 | AD ( *n* = 6)  PIH ( *n* = 4)  BN (*n* = 8) | Non-ablative: 1550 nm (Fraxel re:store); Ablative: 10,600 nm (Fraxel re:pair). | Non-ablative (AD/PIH): 1550 nm (Fraxel re:store); 15 mJ/MTZ; 8 passes; 14–20% coverage; 4–5 sessions; EMLA 2h; cooling.  Ablative (BN): 10,600 nm (Fraxel re:pair); 10 mJ/MTZ; 4 passes; 35–45% coverage; 3 sessions; EMLA 2h. | Intermittent topical bleaching: hydroquinone 5%, tretinoin 0.05%, triamcinolone 0.1% applied once daily from week 2 post-laser until next session. | Two separate randomized controlled observer-blinded split-lesion studies (reported together for histologic comparison) | 6/18 patients (6 AD*, 4 PIH, 8 BN); all completed treatment and biopsy | Non-ablative FLT not effective for AD or PIH. Ablative FLT moderately effective in some BN patients (reported separately). No clinical signs of scarring or fibrosis observed. | Non-ablative (AD/PIH, n=10): No MTZ evidence at 3 months; normal rete ridges; no fibrosis; no differences in collagen deposition. Ablative (BN, n=8): Elongated blunted rete ridges with basal hyperpigmentation; smooth muscle hypertrophy; dermal fibrosis in 4/8 (50%) at 3 months (score 2); significant difference vs. non-ablative (p < 0.05). | Not systematically reported for this histologic substudy. Clinical outcomes and satisfaction reported separately. No PROs specifically for fibrosis. | No clinical signs of scarring or fibrosis visible. Laser-induced PIH was a frequently observed side effect (reported in separate publications). | Non-randomized allocation by condition; no pretreatment biopsies; small sample; H&E may underestimate fibrosis; 3-month follow-up insufficient for persistence; different laser parameters between groups limit attribution. | Ablative FLT induced fibrosis in 50%; non-ablative did not. Unclear if remodeling or scarring. | Laser equipment and disposables provided by B&Co Laser Medico and Solta Medical Inc. exclusively for this study; no role in study design, conduct, data collection, analysis, interpretation, or manuscript preparation/review/approval. Authors report no conflicts of interest. |
| Wolfshohl JA, et al., 2016 | EDP | 1550 nm non-ablative (Fraxel). | 1550 nm non-ablative (Fraxel); spot 15 mm; fluence 5–9 mJ/MTZ; level 5 (14% coverage); 8 passes; 5 sessions at 4–6 week intervals; 4.65–4.91 kJ/session; depth 413–528 μm; 4% lidocaine; cooling; endpoint: mild erythema/edema. | Topical tacrolimus 0.1% once daily (reduced to every other day due to burning); discontinued post-laser. | Case report | 1 patient (35-year-old female, Fitzpatrick skin type IV) | >75% improvement at 3-month follow-up; maintained at 8 months without continued tacrolimus. | Baseline biopsy (2003): pigment incontinence, no macular amyloid (EDP). No post-treatment biopsy. | Patient "very satisfied." Mild temporary burning with tacrolimus (reduced by decreasing frequency). | No post-inflammatory pigmentary alteration or adverse effects occurred throughout the treatment course. | Single case; no control group; tacrolimus and prior treatments confound laser effect; no objective measures or post-treatment histopathology; durability/recurrence uncertain. | 1550 nm FLT + tacrolimus potential option for EDP. Further studies needed. | No external funding. Paul M. Friedman, MD, is a member of the Solta, Inc. Advisory Board; this relationship did not influence study design, conduct, or reporting. Other authors declare no conflicts of interest. |
| Kim SM, et al., 2020 | Riehl's melanosis (pigmented contact dermatitis) | 1927 nm non-ablative TFL (LASEMD™). | 1927 nm non-ablative TFL (LASEMD™); 5W; 10–20 mJ; dynamic/random mode; 3–5 passes; 3–7 sessions at 1-month intervals; topical anesthesia. | Prior failed: Kligman's formula, IPL, QS-Nd:YAG, PDL. Oral prednisolone (10 mg/day) for 2–8 weeks pre-laser in 5 patients with active inflammation. No adjunctive during TFL. | Retrospective case series | 9 patients (8 female, 1 male; aged 44–65 years; Fitzpatrick skin types III–IV); 7 facial lesions, 2 neck lesions | DPASI significantly decreased (9.55 → 5.25). Quartile grading: 6/9 (67%) 51–75% improvement; 1/9 (11%) 76–100%; 2/9 (22%) 26–50%. Melanin index decreased in 2 patients. | Case 1: Pre-treatment: basal vacuolization, lymphocytic infiltration, melanophages. Post-treatment (7 sessions): marked decrease in epidermal melanin and dermal melanophages. | 8/9 "very satisfied" or "satisfied"; 1/9 "slightly satisfied." | Transient mild erythema (faded within 3 days); no scarring or PIH reported. | Small retrospective series (*n* = 9); single center; variable protocols; objective measures/histology only for subset; oral prednisolone confounder; long-term durability not assessed. | 1927 nm TFL safe/effective for Riehl's melanosis; shallow penetration minimizes PIH risk. | No specific funding sources declared. Study conducted as part of routine clinical practice at Severance Hospital, Yonsei University College of Medicine. Authors report no conflicts of interest. |
| Jung CJ, et al., 2023 | Linear lichen planus pigmentosus with pigmentary and atrophic sequelae | 1927 nm non-ablative (Fraxel SR). | 1927 nm non-ablative (Fraxel SR); 60 mJ/MTZ; 304 MTZ/cm²; 7 sessions at 8-week intervals. | Topical tacrolimus 0.1%; intralesional PDRN 1 mL every 8 weeks. | Case report (single case within a case series of two patients) | 1 patient (46-year-old Korean female, Fitzpatrick III) | Improvement in hyperpigmentation with mild residual atrophy after 7 treatments. | Pre-treatment: atrophic epidermis, focal basal vacuolar degeneration, perivascular lymphohistiocytic infiltrates, melanin incontinence. No post-treatment biopsy. | Not reported. | No adverse events or aggravation reported. | Two cases; retrospective; no control/standardized protocol; combination therapy prevents isolating laser effect; different laser modalities used; short follow-up; single-center Korean population. | 1927 nm FLT + PDRN + tacrolimus effective for linear LPP; PDRN offers triple mechanism. | No funding source declared. Authors have nothing to disclose. |
| Murray TN, et al., 2023 | LPP-like drug reaction (hydrochlorothiazide and amlodipine-induced) | 1550 nm non-ablative (Fraxel DUAL). | 1550 nm erbium-doped (Fraxel DUAL); spot 15 mm; fluence 8–13 mJ/MTZ; level 5 (14% coverage); 8 passes; 5 sessions at 4–6 weeks + 3 at 3–6 months (total 8); 1.98–3.89 kJ/session; depth ~500–634 μm; topical anesthesia (benzocaine/lidocaine/tetracaine); cooling; endpoint: mild erythema/edema. | Cysteamine 5% cream once daily for 6 months, then 2×/week maintenance. Prior failed: betamethasone, tacrolimus, triamcinolone, doxycycline, hydroquinone 4%. | Case report | 1 patient (70-year-old female, Fitzpatrick skin type V) | >80% improvement at 3-month follow-up after 7 treatments; continual improvement with each session. | Biopsy 1 (forehead): lichenoid dermatitis with eosinophils, interface changes, pigment incontinence (drug-induced). Biopsy 2 (neck): compatible with LPP or resolving lichenoid drug eruption. No post-treatment biopsy. | Patient endorsed significant satisfaction with degree of improvement. | No adverse events reported. | Single case; no control; combination therapy confounds laser effect; prior tacrolimus may confound; short follow-up; single patient (skin type V) limits generalizability; variable parameters. | 1550 nm FLT + cysteamine effective for LPP-like drug reaction (>80%). First report. | No specific funding sources declared. Authors have indicated no significant interest with commercial supporters. |
| Garg S, et al., 2024 | ADMH – including LPP, EDP, AD, and Riehl's melanosis | Sequential (*Alma Harmony XL^®^*): QS 1064 nm (S1–5) → Er:YAG 2940 nm (S6–7) → hybrid 1570 nm + CO₂ (resistant). | Sequential (Alma Harmony XL): S1–3: Pixel QS 1064 nm (1200 mJ, 4–6 passes); S4–5: QS 1064 nm (1000 mJ, 3–5 mm spot, 1–2 passes); S6–7: Pixel Er:YAG 2940 nm (1200 mJ/pass, 5 passes, long pulse); Resistant: hybrid 1570 nm + CO₂ (parameters not specified). 3-week intervals; mean 5.9 ± 1.4 sessions. | Standard protocol: nutritional correction, sunscreen, emollients, antibiotic-steroid cream for 2 days post-procedure. No ADMH-specific adjunctive therapy. | Retrospective case series (single-centre experience) | 11 patients (Fitzpatrick skin types III–IV); Age: 21.4 ± 5.2 years (mean ± SD) | Physician assessment score: 3.81 ± 1.33 (mean ± SD). Breakdown: Excellent (>90%): 4/11 (36.3%); Good (75–90%): 4/11 (36.3%); Moderate (50–74%): 1/11 (9%); Mild (25–49%): 1/11 (9%); None (<25%): 1/11 (9%). Mean sessions: 5.9 ± 1.4. Follow-up: 9.1 ± 2.1 months. | Not reported for the ADMH cohort. | Patient feedback (Likert 0–10): 5.9 ± 1.4 (mean ± SD). | Transient and treatable side effects (PIH, acneiform eruptions) mentioned generally; not specified for ADMH subgroup. | Retrospective; no control; subjective assessments (no objective tools); no statistical analysis per group; multi-laser protocol limits applicability; Indian population (III–IV) limits generalizability; no long-term durability data. | Sequential multi-laser protocol effective for ADMH (72.6% >75% improvement) in Indian patients. | No external funding received. Authors declare no conflicts of interest. |

Note. Summary of included studies on energy-based devices for acquired dermal macular hyperpigmentation (ADMH). Studies are organized chronologically by year of publication; comprehensive study details are provided in S6 Table. Abbreviations: AD, ashy dermatosis; ADMH, acquired dermal macular hyperpigmentation; BN, Becker's nevus; DPASI, dermal pigmentation area and severity index; EDP, erythema dyschromicum perstans; Er:YAG, erbium-doped yttrium aluminum garnet; H&E, hematoxylin and eosin staining; FLT, fractional laser therapy; IPL, intense pulsed light; LPP, lichen planus pigmentosus; MTZ, microscopic treatment zone; Nd:YAG, neodymium-doped yttrium aluminum garnet; PDRN, polydeoxyribonucleotide; PDL, pulsed dye laser; PhGA, Physician's Global Assessment; PIH, post-inflammatory hyperpigmentation; QS, quality-switched; TFL, thulium fiber laser; VAS, visual analogue scale. Note: The asterisk (*) indicates that Kroon et al. and Wind et al. share a subset of six patients with ashy dermatosis (erythema dyschromicum perstans); after accounting for this overlap, the total number of unique patients across all studies is 31 (rather than the cumulative total of 37).
