# Supplementary material for: Non-ablative dermal remodeling lasers in acquired dermal macular hyperpigmentation: A scoping review
Source: PLoS One. 2026 Sep 28;21(9):e0359552. doi: 10.1371/journal.pone.0359552 (PMC13618957; doi:10.1371/journal.pone.0359552)
Supplement: Supplementary file 8 [file pone.0359552.s010.docx]

**Dear Dr. Gisele Viana de Oliveira and Reviewers,**

We sincerely thank the Academic Editor and the reviewers for their thoughtful and constructive comments on our manuscript. We appreciate the time and expertise invested in evaluating our work, and we are grateful for the positive recognition of the clinical relevance and comprehensive nature of our scoping review.

We have carefully addressed all the points raised and have revised the manuscript accordingly. Below we provide a point-by-point response to each comment. All changes in the revised manuscript have been highlighted using track changes for easy identification

**RESPONSES TO EDITOR COMMENTS**

**Comment 1:** Consistency of study-selection numbers

There are discrepancies in the number of records reported across the manuscript. The Abstract reports 174 identified records, whereas the Results report 193 records identified through electronic database searches, and the Supporting Information refers to 1,267 records retrieved from all sources. These numbers should be reconciled and clearly explained.

Response: We thank the Editor for identifying this discrepancy. We have now carefully reviewed our search records and revised the manuscript to ensure consistency across all sections.

As clarified in the revised manuscript, the numbers are now presented as follows:

1,267 total records identified from all sources (193 from electronic databases + 1,074 from citation tracking)

193 records identified through electronic database searches, which were carried forward for deduplication and formal screening

1,074 records identified through citation tracking of reviews and included studies, none of which met eligibility criteria during pre-screening

7 studies included in the final review

We have revised the Abstract, Results section, PRISMA-ScR flow diagram, and supplementary materials to present these numbers consistently. The Abstract now reflects the correct sequential flow of the study selection process, and the Results section provides a detailed explanation of how the 1,267 total records were derived.

**Comment 2:** Interpretation of randomized versus observational evidence

The hypothesis that disease activity, timing of laser treatment, and concomitant immunomodulation may explain the discrepancy between negative randomized trials and favorable observational reports is clinically interesting and biologically plausible. However, the available evidence does not support a causal conclusion. In particular, the statement that the "critical distinction" was continuous tacrolimus treatment and intervention during a stable disease phase appears too definitive. These factors should instead be presented as hypothesis-generating explanations requiring prospective evaluation.

Response: We fully agree with this important distinction. We have revised the manuscript throughout to ensure that our interpretation is presented as a hypothesis-generating explanation rather than a definitive causal conclusion.

Specifically:

We have removed definitive language such as "the critical distinction was" and replaced it with cautious phrasing in the Discussion section.

We have explicitly stated that this interpretation is consistent with available observations but should be considered hypothesis-generating and warranting prospective evaluation.

We have added language emphasizing that the available evidence does not permit causal inferences regarding the role of disease activity, timing of laser treatment, or concomitant immunomodulation.

We believe these revisions appropriately balance the biological plausibility of the hypothesis with the limitations of the available evidence.

**Comment 3:** Independent effect of laser therapy

Most favorable observational studies included concomitant therapies such as tacrolimus, systemic corticosteroids, cysteamine, PDRN, or multimodal laser protocols. Consequently, the independent contribution of NADRL cannot be established. Although this limitation is acknowledged, it should be emphasized consistently when interpreting positive studies, avoiding language that could imply established efficacy.

Response: We thank the Editor for this important observation. We have carefully revised the manuscript to consistently emphasize this limitation throughout the Results and Discussion sections.

Changes made:

In the Results section, we now explicitly highlight that most observational studies used combination regimens, precluding attribution of outcomes to laser therapy alone.

In the Discussion, we have added explicit statements emphasizing that the independent contribution of NADRL cannot be established from the current evidence.

The Conclusion now prominently states that "the frequent use of concomitant therapies in positive reports, combined with the lack of controlled studies isolating the laser effect, further underscores that any observed benefits cannot be attributed to the laser alone."

**Comment 4:** Dermal remodeling as a proposed mechanism

The conceptual focus on dermal remodeling is interesting, but there is an important gap between the proposed mechanism and the endpoints actually evaluated. Most included studies assessed pigmentation rather than objective structural, histological, or molecular evidence of dermal remodeling. The distinction between a biologically plausible rationale and a demonstrated therapeutic mechanism should therefore remain explicit throughout the manuscript.

Response: We agree that this is a critical distinction that must be maintained throughout the manuscript. We have revised the text to clearly differentiate between the biological rationale for NADRL use (dermal remodeling) and the clinical endpoints actually measured in the included studies (pigmentation).

Changes made:

The Introduction now ends with the explicit statement: "However, this remodeling hypothesis remains unproven in ADMH."

The Discussion now clearly states that "although the biological rationale for dermal remodeling in ADMH is plausible, most studies evaluated only pigmentary outcomes rather than biological or structural markers of dermal remodeling, leaving the proposed mechanism largely untested."

We have added a dedicated paragraph discussing the need for mechanistic endpoints in future studies, including histology, molecular biomarkers, and non-invasive imaging.

**Comment 5:** Disease activity and patient stratification

The distinction between active inflammatory ADMH and stable residual pigmentation may be clinically important. However, disease activity was not systematically defined or prospectively evaluated in the included studies. Conclusions regarding differential response according to disease stage should therefore remain cautious, and evidence-based findings should be clearly distinguished from hypotheses for future research.

Response: We appreciate this important clarification. We have revised the manuscript to ensure that our discussion of disease activity is presented as a hypothesis for future research rather than an evidence-based finding.

Changes made:

We have explicitly stated that "disease activity was not systematically evaluated in the included studies."

We have revised the language to present the distinction between active inflammatory ADMH and stable residual pigmentation as "a hypothesis that extrapolates the recognized importance of disease stage when using lasers in ADMH."

We have added a recommendation for future studies to prospectively stratify patients according to predefined disease activity categories.

**Comment 6:** Safety and darker phototypes

Given that ADMH frequently affects darker skin phototypes and that laser-induced post-inflammatory hyperpigmentation was reported in one randomized trial, the limited representation and systematic assessment of Fitzpatrick V–VI should be emphasized. The theoretical safety advantage of water-targeting lasers over pigment-targeting devices should not be presented as an established clinical advantage in ADMH.

Response: We thank the Editor for this important safety consideration. We agree that the limited representation of Fitzpatrick skin types V–VI and the theoretical nature of the safety advantage must be clearly emphasized.

In the revised manuscript, we have strengthened the discussion on phototype representation by adding a specific observation to the existing paragraph on safety limitations: "Of note, only one study included a single Fitzpatrick V patient [69], and none included Fitzpatrick VI." This addition highlights the near-total absence of darker phototypes in the current evidence base.

The existing text already emphasized that adverse events and PIH were not systematically assessed, that objective pigment measurements were infrequently used, and that no study specifically evaluated Fitzpatrick skin types V–VI despite these populations being at the greatest risk of pigmentary complications. We have maintained these statements and preserved the recommendation that future prospective studies should incorporate standardized adverse-event reporting, objective pigment assessment, and adequate representation of darker phototypes.

We have also ensured that the theoretical safety advantage of water-targeting lasers over pigment-targeting devices is not presented as an established clinical benefit, but rather as a hypothesis requiring prospective validation. The safety profile of NADRL in ADMH remains incompletely characterized, particularly in darker phototypes.

**Minor Comments**

**Comment:** Editorial revision and terminology

The manuscript would benefit from careful editorial revision to correct minor typographical inconsistencies and standardize terminology and abbreviations throughout the manuscript and supplementary material.

Response: We have carefully reviewed the entire manuscript and supplementary materials to correct typographical errors, standardize terminology, and ensure consistent use of abbreviations throughout. The revised manuscript has been thoroughly proofread.

**RESPONSES TO REVIEWER COMMENTS**

**Reviewer #1**

We thank Reviewer #1 for the positive evaluation and constructive feedback. We are pleased that the reviewer found our data synthesis appropriate and our conclusions appropriately cautious. The reviewer's recognition of our work as a comprehensive review that avoids exaggerated claims is greatly appreciated.

We have addressed the methodological consistency issues as outlined in our responses to the Editor's comments above.

**Reviewer #2**

We thank Reviewer #2 for the detailed and insightful review. The reviewer's thorough evaluation and constructive suggestions have substantially strengthened our manuscript. We address each point below.

**Comment 1:** Consistency of study-selection numbers

There are discrepancies in the number of records reported across the manuscript. The Abstract reports 174 identified records, whereas the Results report 193 records identified through electronic database searches, and the Supporting Information refers to 1,267 records retrieved from all sources.

Response: We have carefully reconciled the study selection numbers as described in our response to the Editor's comments (Comment 1). The Abstract, Methods, Results, PRISMA-ScR flow diagram, and supplementary materials now present fully consistent numbers.

The revised Results section now clearly explains:

193 records identified from electronic database searches

1,074 records identified through citation tracking

1,267 total records from all sources

7 studies included in the final review

All discrepancies have been resolved.

**Comment 2:** Interpretation of randomized versus observational evidence

The hypothesis that disease activity, timing of laser treatment, and concomitant immunomodulation may explain the discrepancy between negative randomized trials and favorable observational reports is clinically interesting and biologically plausible. However, the available evidence does not support a causal conclusion. In particular, the statement that the "critical distinction" was continuous tacrolimus treatment and intervention during a stable disease phase appears too definitive.

Response: We agree with the reviewer's assessment. We have revised the manuscript throughout to present these explanations as hypothesis-generating rather than definitive conclusions. The language has been softened to reflect the exploratory nature of these observations, and we have explicitly stated that prospective evaluation is needed.

The Discussion section now reads: "This suggests that the key difference may lie in the concomitant use of continuous topical tacrolimus and application during a stable disease phase rather than active inflammation; a hypothesis that extrapolates the recognized importance of disease stage when using lasers in ADMH. Although disease activity was not systematically evaluated in the included studies, this interpretation is consistent with the available observations and should be considered hypothesis-generating, warranting prospective evaluation, precluding attribution to the laser alone."

**Comment 3:** Independent effect of laser therapy

Most favorable observational studies included concomitant therapies such as tacrolimus, systemic corticosteroids, cysteamine, PDRN, or multimodal laser protocols. Consequently, the independent contribution of NADRL cannot be established. Although this limitation is acknowledged, it should be emphasized consistently.

Response: We thank the reviewer for this important emphasis. We have revised the Results and Discussion sections to consistently highlight this limitation whenever interpreting positive studies. Language that could imply established efficacy has been avoided throughout. The Conclusion now explicitly states: "The frequent use of concomitant therapies in positive reports, combined with the lack of controlled studies isolating the laser effect, further underscores that any observed benefits cannot be attributed to the laser alone."

**Comment 4:** Dermal remodeling as a proposed mechanism

The conceptual focus on dermal remodeling is interesting, but there is an important gap between the proposed mechanism and the endpoints actually evaluated. Most included studies assessed pigmentation rather than objective structural, histological, or molecular evidence of dermal remodeling.

Response: We have revised the manuscript to clearly distinguish between the biological rationale (dermal remodeling) and the endpoints actually measured (pigmentation). This distinction is now explicit throughout the manuscript, and we have framed dermal remodeling as a plausible mechanism requiring objective validation in future studies. The Introduction now ends with "However, this remodeling hypothesis remains unproven in ADMH."

**Comment 5:** Disease activity and patient stratification

Disease activity was not systematically defined or prospectively evaluated in the included studies. Conclusions regarding differential response according to disease stage should therefore remain cautious.

Response: We have revised the relevant sections to reflect this limitation. The distinction between active inflammatory ADMH and stable residual pigmentation is now presented as a hypothesis for future research rather than an evidence-based conclusion. We have explicitly stated that disease activity was not systematically evaluated in the included studies.

**Comment 6:** Safety and darker phototypes

We thank the reviewer for this important safety consideration. We agree that the limited representation of Fitzpatrick skin types V–VI and the theoretical nature of the safety advantage of water-targeting lasers must be clearly emphasized.

In the revised manuscript, we have strengthened this discussion by adding a specific observation: "Of note, only one study included a single Fitzpatrick V patient [69], and none included Fitzpatrick VI." This addition explicitly highlights the near-total absence of darker phototypes in the current evidence base.

Additionally, we have maintained the existing statements that adverse events and PIH were not systematically assessed, and that the safety profile of NADRL in ADMH remains incompletely characterized. We have also preserved the recommendation that future prospective studies should incorporate adequate representation of darker phototypes and standardized adverse-event reporting.

We believe these revisions adequately address the reviewer's concern regarding the limited phototype representation and the need to avoid presenting theoretical safety advantages as established clinical benefits.

**Minor Comments**

The manuscript would benefit from careful editorial revision to correct minor typographical inconsistencies and standardize terminology.

Response: We have carefully edited the entire manuscript and supplementary materials to address all typographical and terminological issues.

**SUMMARY OF MAJOR REVISIONS**

**Study selection numbers:** Fully reconciled across Abstract, Methods, Results, PRISMA-ScR flow diagram, and supplementary materials. The relationship between 193 database records, 1,074 citation-tracking records, and 1,267 total records is now clearly explained.

**Interpretation of findings:** Language revised throughout to present hypotheses as hypothesis-generating rather than definitive conclusions. Definitive language such as "the critical distinction was" has been replaced with cautious phrasing.

**Independent effect of laser therapy:** Limitation emphasized consistently throughout Results and Discussion. The Conclusion now explicitly states that observed benefits cannot be attributed to the laser alone.

**Dermal remodeling mechanism:** Clear distinction maintained between biological rationale and demonstrated therapeutic mechanism. The Introduction now explicitly states that the remodeling hypothesis remains unproven in ADMH.

**Disease activity:** Presented as a hypothesis for future research requiring prospective evaluation. We explicitly acknowledge that disease activity was not systematically evaluated in the included studies.

**Safety and phototypes:** Emphasis added on limited representation of Fitzpatrick V–VI. The theoretical safety advantage is now clearly framed as unproven in ADMH.

**Editorial revision:** Thorough language and terminology review completed throughout the manuscript and supplementary materials.

We believe that these revisions have substantially strengthened the methodological rigor and interpretative clarity of our manuscript. We are grateful for the opportunity to revise and hope that the revised version meets the standards for publication in PLOS One.

We look forward to your further evaluation.

Sincerely,

Virgilio Blandón, MD

Corresponding Author

On behalf of all coauthors
