# Supplementary material for: Non-ablative dermal remodeling lasers in acquired dermal macular hyperpigmentation: A scoping review
Source: PLoS One. 2026 Sep 28;21(9):e0359552. doi: 10.1371/journal.pone.0359552 (PMC13618957; doi:10.1371/journal.pone.0359552)
Supplement: Peer review history

# Peer review: Decision Letter 0

**DOI:** 10.1371/journal.pone.0359552.r001

**Authors:** Gisele Viana de Oliveira

23 Aug 2026

Dear Dr. Blandon,

Thank you for submitting your manuscript to PLOS One. After careful consideration, we feel that it has merit but does not fully meet PLOS One’s publication criteria as it currently stands. Therefore, we invite you to submit a revised version of the manuscript that addresses the points raised during the review process.

Please submit your revised manuscript by Oct 22 2026 11:59PM. If you will need more time than this to complete your revisions, please reply to this message or contact the journal office at plosone@plos.org. When you're ready to submit your revision, log on to https://www.editorialmanager.com/pone/ and select the 'Submissions Needing Revision' folder to locate your manuscript file.

- A letter that responds to each point raised by the academic editor and reviewer(s). You should upload this letter as a separate file labeled 'Response to Reviewers'.
- A marked-up copy of your manuscript that highlights changes made to the original version. You should upload this as a separate file labeled 'Revised Manuscript with Track Changes'.
- An unmarked version of your revised paper without tracked changes. You should upload this as a separate file labeled 'Manuscript'.

If you would like to make changes to your financial disclosure, please include your updated statement in your cover letter. Guidelines for resubmitting your figure files are available below the reviewer comments at the end of this letter.

If applicable, we recommend that you deposit your laboratory protocols in protocols.io to enhance the reproducibility of your results. Protocols.io assigns your protocol its own identifier (DOI) so that it can be cited independently in the future. For instructions see: https://journals.plos.org/plosone/s/submission-guidelines#loc-laboratory-protocols. Additionally, PLOS One offers an option for publishing peer-reviewed Lab Protocol articles, which describe protocols hosted on protocols.io. Read more information on sharing protocols at https://plos.org/protocols?utm_medium=editorial-email&utm_source=authorletters&utm_campaign=protocols.

As the corresponding author, your ORCID iD is verified in the submission system and will appear in the published article. PLOS supports the use of ORCID, and we encourage all coauthors to register for an ORCID iD and use it as well. Please encourage your coauthors to verify their ORCID iD within the submission system before final acceptance, as unverified ORCID iDs will not appear in the published article. *Only*  the individual author can complete the verification step; PLOS staff *cannot*  verify ORCID iDs on behalf of authors.

We look forward to receiving your revised manuscript.

Kind regards,

Gisele Viana de Oliveira

Academic Editor

PLOS One

**Journal Requirements:**

When submitting your revision, we need you to address these additional requirements.

1. Please ensure that your manuscript meets PLOS ONE's style requirements, including those for file naming. The PLOS ONE style templates can be found at

https://journals.plos.org/plosone/s/file?id=wjVg/PLOSOne_formatting_sample_main_body.pdf and

https://journals.plos.org/plosone/s/file?id=ba62/PLOSOne_formatting_sample_title_authors_affiliations.pdf

2. If the reviewer comments include a recommendation to cite specific previously published works, please review and evaluate these publications to determine whether they are relevant and should be cited. There is no requirement to cite these works unless the editor has indicated otherwise.

**Additional Editor Comments:**

This manuscript addresses a clinically relevant and insufficiently explored topic: the use of non-ablative dermal remodeling lasers (NADRL) in acquired dermal macular hyperpigmentation (ADMH). The review is comprehensive, the biological rationale is interesting, and the overall conclusion—that current evidence is limited, heterogeneous, and insufficient to establish the independent efficacy of these devices—is appropriately cautious and generally supported by the available literature.

However, several important issues should be addressed before publication.

Major comments

Consistency of study-selection numbers. There are discrepancies in the number of records reported across the manuscript. The Abstract reports 174 identified records, whereas the Results report 193 records identified through electronic database searches, and the Supporting Information refers to 1,267 records retrieved from all sources. These numbers should be reconciled and clearly explained. The Abstract, Methods, Results, PRISMA-ScR flow diagram, and supplementary material should present a fully consistent study-selection process.

Interpretation of randomized versus observational evidence. The hypothesis that disease activity, timing of laser treatment, and concomitant immunomodulation may explain the discrepancy between negative randomized trials and favorable observational reports is clinically interesting and biologically plausible. However, the available evidence does not support a causal conclusion. In particular, the statement that the “critical distinction” was continuous tacrolimus treatment and intervention during a stable disease phase appears too definitive. These factors should instead be presented as hypothesis-generating explanations requiring prospective evaluation.

Independent effect of laser therapy. Most favorable observational studies included concomitant therapies such as tacrolimus, systemic corticosteroids, cysteamine, PDRN, or multimodal laser protocols. Consequently, the independent contribution of NADRL cannot be established. Although this limitation is acknowledged, it should be emphasized consistently when interpreting positive studies, avoiding language that could imply established efficacy.

Dermal remodeling as a proposed mechanism. The conceptual focus on dermal remodeling is interesting, but there is an important gap between the proposed mechanism and the endpoints actually evaluated. Most included studies assessed pigmentation rather than objective structural, histological, or molecular evidence of dermal remodeling. The distinction between a biologically plausible rationale and a demonstrated therapeutic mechanism should therefore remain explicit throughout the manuscript.

Disease activity and patient stratification. The distinction between active inflammatory ADMH and stable residual pigmentation may be clinically important. However, disease activity was not systematically defined or prospectively evaluated in the included studies. Conclusions regarding differential response according to disease stage should therefore remain cautious, and evidence-based findings should be clearly distinguished from hypotheses for future research.

Safety and darker phototypes. Given that ADMH frequently affects darker skin phototypes and that laser-induced post-inflammatory hyperpigmentation was reported in one randomized trial, the limited representation and systematic assessment of Fitzpatrick V–VI should be emphasized. The theoretical safety advantage of water-targeting lasers over pigment-targeting devices should not be presented as an established clinical advantage in ADMH.

Minor comments

The manuscript would benefit from careful editorial revision to correct minor typographical inconsistencies and standardize terminology and abbreviations throughout the manuscript and supplementary material.

Overall, this is a potentially valuable scoping review addressing an important therapeutic challenge. The principal conclusions are appropriately cautious. However, methodological consistency and a clearer separation between demonstrated findings and biologically plausible hypotheses are necessary to strengthen the manuscript

[Note: HTML markup is below. Please do not edit.]

Reviewers' comments:

Reviewer's Responses to Questions

**Comments to the Author**

1. Is the manuscript technically sound, and do the data support the conclusions?

Reviewer #1: Yes

Reviewer #2: Yes

**********

2. Has the statistical analysis been performed appropriately and rigorously?

Reviewer #1: Yes

Reviewer #2: N/A

**********

3. Have the authors made all data underlying the findings in their manuscript fully available?

The PLOS Data policy

Reviewer #1: Yes

Reviewer #2: Yes

**********

4. Is the manuscript presented in an intelligible fashion and written in standard English?

Reviewer #1: Yes

Reviewer #2: Yes

**********

Reviewer #1: yes, The authors have performed the data synthesis appropriately by opting for a narrative and descriptive approach rather than a quantitative meta-analysis

The authors have stated that all data underlying their findings are fully available without restriction

According to the Data Availability Statement in the manuscript, the information is provided as follows:

Location: All data generated or analyzed during the scoping review are included within the published article itself and its supplementary information files

Contents: The available data includes search strategies, screening decisions, exclusion codes, data extraction forms, risk of bias assessments, and the completed PRISMA-ScR checklist

Specific Files: These materials are labeled as supporting information S1–S5 Tables and S1–S2 Texts

Additional Details: The complete screening process (including deduplication records, detailed exclusion decisions, and citation tracking results) is provided in S1 Appendix

In conclusion, the manuscript avoids making exaggerated claims and limits itself to noting that these devices remain investigational, recommending more standardized future research with clear biological endpoints

Reviewer #2: This manuscript addresses a clinically relevant and insufficiently explored topic: the use of non-ablative dermal remodeling lasers (NADRL) in acquired dermal macular hyperpigmentation (ADMH). The review is comprehensive, the biological rationale is interesting, and the overall conclusion—that current evidence is limited, heterogeneous, and insufficient to establish the independent efficacy of these devices—is appropriately cautious and generally supported by the available literature.

However, several important issues should be addressed before publication.

Major comments

Consistency of study-selection numbers. There are discrepancies in the number of records reported across the manuscript. The Abstract reports 174 identified records, whereas the Results report 193 records identified through electronic database searches, and the Supporting Information refers to 1,267 records retrieved from all sources. These numbers should be reconciled and clearly explained. The Abstract, Methods, Results, PRISMA-ScR flow diagram, and supplementary material should present a fully consistent study-selection process.

Interpretation of randomized versus observational evidence. The hypothesis that disease activity, timing of laser treatment, and concomitant immunomodulation may explain the discrepancy between negative randomized trials and favorable observational reports is clinically interesting and biologically plausible. However, the available evidence does not support a causal conclusion. In particular, the statement that the “critical distinction” was continuous tacrolimus treatment and intervention during a stable disease phase appears too definitive. These factors should instead be presented as hypothesis-generating explanations requiring prospective evaluation.

Independent effect of laser therapy. Most favorable observational studies included concomitant therapies such as tacrolimus, systemic corticosteroids, cysteamine, PDRN, or multimodal laser protocols. Consequently, the independent contribution of NADRL cannot be established. Although this limitation is acknowledged, it should be emphasized consistently when interpreting positive studies, avoiding language that could imply established efficacy.

Dermal remodeling as a proposed mechanism. The conceptual focus on dermal remodeling is interesting, but there is an important gap between the proposed mechanism and the endpoints actually evaluated. Most included studies assessed pigmentation rather than objective structural, histological, or molecular evidence of dermal remodeling. The distinction between a biologically plausible rationale and a demonstrated therapeutic mechanism should therefore remain explicit throughout the manuscript.

Disease activity and patient stratification. The distinction between active inflammatory ADMH and stable residual pigmentation may be clinically important. However, disease activity was not systematically defined or prospectively evaluated in the included studies. Conclusions regarding differential response according to disease stage should therefore remain cautious, and evidence-based findings should be clearly distinguished from hypotheses for future research.

Safety and darker phototypes. Given that ADMH frequently affects darker skin phototypes and that laser-induced post-inflammatory hyperpigmentation was reported in one randomized trial, the limited representation and systematic assessment of Fitzpatrick V–VI should be emphasized. The theoretical safety advantage of water-targeting lasers over pigment-targeting devices should not be presented as an established clinical advantage in ADMH.

Minor comments

The manuscript would benefit from careful editorial revision to correct minor typographical inconsistencies and standardize terminology and abbreviations throughout the manuscript and supplementary material.

Overall, this is a potentially valuable scoping review addressing an important therapeutic challenge. The principal conclusions are appropriately cautious. However, methodological consistency and a clearer separation between demonstrated findings and biologically plausible hypotheses are necessary to strengthen the manuscript.

**********

what does this mean?). If published, this will include your full peer review and any attached files.

If you choose “no”, your identity will remain anonymous but your review may still be made public.

**Do you want your identity to be public for this peer review?**  For information about this choice, including consent withdrawal, please see our Privacy Policy

Reviewer #1: **Yes:**  patricia apt druck

Reviewer #2: No

**********

[NOTE: If reviewer comments were submitted as an attachment file, they will be attached to this email and accessible via the submission site. Please log into your account, locate the manuscript record, and check for the action link "View Attachments". If this link does not appear, there are no attachment files.]

To ensure your figures meet our technical requirements, please review our figure guidelines: https://journals.plos.org/plosone/s/figures

You may also use PLOS’s free figure tool, NAAS, to help you prepare publication quality figures: https://journals.plos.org/plosone/s/figures#loc-tools-for-figure-preparation.

NAAS will assess whether your figures meet our technical requirements by comparing each figure against our figure specifications.

# Author comment: Author response to Decision Letter 1

**DOI:** 10.1371/journal.pone.0359552.r002

27 Aug 2026

Dear Dr. Gisele Viana de Oliveira and Reviewers,

We sincerely thank the Academic Editor and the reviewers for their thoughtful and constructive comments on our manuscript. We appreciate the time and expertise invested in evaluating our work, and we are grateful for the positive recognition of the clinical relevance and comprehensive nature of our scoping review.

We have carefully addressed all the points raised and have revised the manuscript accordingly. Below we provide a point-by-point response to each comment. All changes in the revised manuscript have been highlighted using track changes for easy identification

RESPONSES TO EDITOR COMMENTS

Comment 1: Consistency of study-selection numbers

There are discrepancies in the number of records reported across the manuscript. The Abstract reports 174 identified records, whereas the Results report 193 records identified through electronic database searches, and the Supporting Information refers to 1,267 records retrieved from all sources. These numbers should be reconciled and clearly explained.

Response: We thank the Editor for identifying this discrepancy. We have now carefully reviewed our search records and revised the manuscript to ensure consistency across all sections.

As clarified in the revised manuscript, the numbers are now presented as follows:

1,267 total records identified from all sources (193 from electronic databases + 1,074 from citation tracking)

193 records identified through electronic database searches, which were carried forward for deduplication and formal screening

1,074 records identified through citation tracking of reviews and included studies, none of which met eligibility criteria during pre-screening

7 studies included in the final review

We have revised the Abstract, Results section, PRISMA-ScR flow diagram, and supplementary materials to present these numbers consistently. The Abstract now reflects the correct sequential flow of the study selection process, and the Results section provides a detailed explanation of how the 1,267 total records were derived.

Comment 2: Interpretation of randomized versus observational evidence

The hypothesis that disease activity, timing of laser treatment, and concomitant immunomodulation may explain the discrepancy between negative randomized trials and favorable observational reports is clinically interesting and biologically plausible. However, the available evidence does not support a causal conclusion. In particular, the statement that the "critical distinction" was continuous tacrolimus treatment and intervention during a stable disease phase appears too definitive. These factors should instead be presented as hypothesis-generating explanations requiring prospective evaluation.

Response: We fully agree with this important distinction. We have revised the manuscript throughout to ensure that our interpretation is presented as a hypothesis-generating explanation rather than a definitive causal conclusion.

Specifically:

We have removed definitive language such as "the critical distinction was" and replaced it with cautious phrasing in the Discussion section.

We have explicitly stated that this interpretation is consistent with available observations but should be considered hypothesis-generating and warranting prospective evaluation.

We have added language emphasizing that the available evidence does not permit causal inferences regarding the role of disease activity, timing of laser treatment, or concomitant immunomodulation.

We believe these revisions appropriately balance the biological plausibility of the hypothesis with the limitations of the available evidence.

Comment 3: Independent effect of laser therapy

Most favorable observational studies included concomitant therapies such as tacrolimus, systemic corticosteroids, cysteamine, PDRN, or multimodal laser protocols. Consequently, the independent contribution of NADRL cannot be established. Although this limitation is acknowledged, it should be emphasized consistently when interpreting positive studies, avoiding language that could imply established efficacy.

Response: We thank the Editor for this important observation. We have carefully revised the manuscript to consistently emphasize this limitation throughout the Results and Discussion sections.

Changes made:

In the Results section, we now explicitly highlight that most observational studies used combination regimens, precluding attribution of outcomes to laser therapy alone.

In the Discussion, we have added explicit statements emphasizing that the independent contribution of NADRL cannot be established from the current evidence.

The Conclusion now prominently states that "the frequent use of concomitant therapies in positive reports, combined with the lack of controlled studies isolating the laser effect, further underscores that any observed benefits cannot be attributed to the laser alone."

Comment 4: Dermal remodeling as a proposed mechanism

The conceptual focus on dermal remodeling is interesting, but there is an important gap between the proposed mechanism and the endpoints actually evaluated. Most included studies assessed pigmentation rather than objective structural, histological, or molecular evidence of dermal remodeling. The distinction between a biologically plausible rationale and a demonstrated therapeutic mechanism should therefore remain explicit throughout the manuscript.

Response: We agree that this is a critical distinction that must be maintained throughout the manuscript. We have revised the text to clearly differentiate between the biological rationale for NADRL use (dermal remodeling) and the clinical endpoints actually measured in the included studies (pigmentation).

Changes made:

The Introduction now ends with the explicit statement: "However, this remodeling hypothesis remains unproven in ADMH."

The Discussion now clearly states that "although the biological rationale for dermal remodeling in ADMH is plausible, most studies evaluated only pigmentary outcomes rather than biological or structural markers of dermal remodeling, leaving the proposed mechanism largely untested."

We have added a dedicated paragraph discussing the need for mechanistic endpoints in future studies, including histology, molecular biomarkers, and non-invasive imaging.

Comment 5: Disease activity and patient stratification

The distinction between active inflammatory ADMH and stable residual pigmentation may be clinically important. However, disease activity was not systematically defined or prospectively evaluated in the included studies. Conclusions regarding differential response according to disease stage should therefore remain cautious, and evidence-based findings should be clearly distinguished from hypotheses for future research.

Response: We appreciate this important clarification. We have revised the manuscript to ensure that our discussion of disease activity is presented as a hypothesis for future research rather than an evidence-based finding.

Changes made:

We have explicitly stated that "disease activity was not systematically evaluated in the included studies."

We have revised the language to present the distinction between active inflammatory ADMH and stable residual pigmentation as "a hypothesis that extrapolates the recognized importance of disease stage when using lasers in ADMH."

We have added a recommendation for future studies to prospectively stratify patients according to predefined disease activity categories.

Comment 6: Safety and darker phototypes

Given that ADMH frequently affects darker skin phototypes and that laser-induced post-inflammatory hyperpigmentation was reported in one randomized trial, the limited representation and systematic assessment of Fitzpatrick V–VI should be emphasized. The theoretical safety advantage of water-targeting lasers over pigment-targeting devices should not be presented as an established clinical advantage in ADMH.

Response: We thank the Editor for this important safety consideration. We agree that the limited representation of Fitzpatrick skin types V–VI and the theoretical nature of the safety advantage must be clearly emphasized.

In the revised manuscript, we have strengthened the discussion on phototype representation by adding a specific observation to the existing paragraph on safety limitations: "Of note, only one study included a single Fitzpatrick V patient [69], and none included Fitzpatrick VI." This addition highlights the near-total absence of darker phototypes in the current evidence base.

The existing text already emphasized that adverse events and PIH were not systematically assessed, that objective pigment measurements were infrequently used, and that no study specifically evaluated Fitzpatrick skin types V–VI despite these populations being at the greatest risk of pigmentary complications. We have maintained these statements and preserved the recommendation that future prospective studies should incorporate standardized adverse-event reporting, objective pigment assessment, and adequate representation of darker phototypes.

We have also ensured that the theoretical safety advantage of water-targeting lasers over pigment-targeting devices is not presented as an established clinical benefit, but rather as a hypothesis requiring prospective validation. The safety profile of NADRL in ADMH remains incompletely characterized, particularly in darker phototypes.

Minor Comments

Comment: Editorial revision and terminology

The manuscript would benefit from careful editorial revision to correct minor typographical inconsistencies and standardize terminology and abbreviations throughout the manuscript and supplementary material.

Response: We have carefully reviewed the entire manuscript and supplementary materials to correct typographical errors, standardize terminology, and ensure consistent use of abbreviations throughout. The revised manuscript has been thoroughly proofread.

RESPONSES TO REVIEWER COMMENTS

Reviewer #1

We thank Reviewer #1 for the positive evaluation and constructive feedback. We are pleased that the reviewer found our data synthesis appropriate and our conclusions appropriately cautious. The reviewer's recognition of our work as a comprehensive review that avoids exaggerated claims is greatly appreciated.

We have addressed the methodological consistency issues as outlined in our responses to the Editor's comments above.

Reviewer #2

We thank Reviewer #2 for the detailed and insightful review. The reviewer's thorough evaluation and constructive suggestions have substantially strengthened our manuscript. We address each point below.

Comment 1: Consistency of study-selection numbers

There are discrepancies in the number of records reported across the manuscript. The Abstract reports 174 identified records, whereas the Results report 193 records identified through electronic database searches, and the Supporting Information refers to 1,267 records retrieved from all sources.

Response: We have carefully reconciled the study selection numbers as described in our response to the Editor's comments (Comment 1). The Abstract, Methods, Results, PRISMA-ScR flow diagram, and supplementary materials now present fully consistent numbers.

The revised Results section now clearly explains:

193 records identified from electronic database searches

1,074 records identified through citation tracking

1,267 total records from all sources

7 studies included in the final review

All discrepancies have been resolved.

Comment 2: Interpretation of randomized versus observational evidence

The hypothesis that disease activity, timing of laser treatment, and concomitant immunomodulation may explain the discrepancy between negative randomized trials and favorable observational reports is clinically interesting and biologically plausible. However, the available evidence does not support a causal conclusion. In particular, the statement that the "critical distinction" was continuous tacrolimus treatment and intervention during a stable disease phase appears too definitive.

Response: We agree with the reviewer's assessment. We have revised the manuscript throughout to present these explanations as hypothesis-generating rather than definitive conclusions. The language has been softened to reflect the exploratory nature of these observations, and we have explicitly stated that prospective evaluation is needed.

The Discussion section now reads: "This suggests that the key difference may lie in the concomitant use of continuous topical tacrolimus and application during a stable disease phase rather than active inflammation; a hypothesis that extrapolates the recognized importance of disease stage when using lasers in ADMH. Although disease activity was not systematically evaluated in the included studies, this interpretation is consistent with the available observations and should be considered hypothesis-generating, warranting prospective evaluation, precluding attribution to the laser alone."

Comment 3: Independent effect of laser therapy

Most favorable observational studies included concomitant therapies such as tacrolimus, systemic corticosteroids, cysteamine, PDRN, or multimodal laser protocols. Consequently, the independent contribution of NADRL cannot be established. Although this limitation is acknowledged, it should be emphasized consistently.

Response: We thank the reviewer for this important emphasis. We have revised the Results and Discussion sections to consistently highlight this limitation whenever interpreting positive studies. Language that could imply established efficacy has been avoided throughout. The Conclusion now explicitly states: "The frequent use of concomitant therapies in positive reports, combined with the lack of controlled studies isolating the laser effect, further underscores that any observed benefits cannot be attributed to the laser alone."

Comment 4: Dermal remodeling as a proposed mechanism

The conceptual focus on dermal remodeling is interesting, but there is an important gap between the proposed mechanism and the endpoints actually evaluated. Most included studies assessed pigmentation rather than objective structural, histological, or molecular evidence of dermal remodeling.

Response: We have revised the manuscript to clearly distinguish between the biological rationale (dermal remodeling) and the endpoints actually measured (pigmentation). This distinction is now explicit throughout the manuscript, and we have framed dermal remodeling as a plausible mechanism requiring objective validation in future studies. The Introduction now ends with "However, this remodeling hypothesis remains unproven in ADMH."

Comment 5: Disease activity and patient stratification

Disease activity was not systematically defined or prospectively evaluated in the included studies. Conclusions regarding differential response according to disease stage should therefore remain cautious.

Response: We have revised the relevant sections to reflect this limitation. The distinction between active inflammatory ADMH and stable residual pigmentation is now presented as a hypothesis for future research rather than an evidence-based conclusion. We have explicitly stated that disease activity was not systematically evaluated in the included studies.

Comment 6: Safety and darker phototypes

We thank the reviewer for this important safety consideration. We agree that the limited representation of Fitzpatrick skin types V–VI and the theoretical nature of the safety advantage of water-targeting lasers must be clearly emphasized.

In the revised manuscript, we have strengthened this discussion by adding a specific observation: "Of note, only one study included a single Fitzpatrick V patient [69], and none included Fitzpatrick VI." This addition explicitly highlights the near-total absence of darker phototypes in the current evidence base.

Additionally, we have maintained the existing statements that adverse events and PIH were not systematically assessed, and that the safety profile of NADRL in ADMH remains incompletely characterized. We have also preserved the recommendation that future prospective studies should incorporate adequate representation of darker phototypes and standardized adverse-event reporting.

We believe these revisions adequately address the reviewer's concern regarding the limited phototype representation and the need to avoid presenting theoretical safety advantages as established clinical benefits.

Minor Comments

The manuscript would benefit from careful editorial revision to correct minor typographical inconsistencies and standardize terminology.

Response: We have carefully edited the entire manuscript and supplementary materials to address all typographical and terminological issues.

SUMMARY OF MAJOR REVISIONS

Study selection numbers: Fully reconciled across Abstract, Methods, Results, PRISMA-ScR flow diagram, and supplementary materials. The relationship between 193 database records, 1,074 citation-tracking records, and 1,267 total records is now clearly explained.

Interpretation of findings: Language revised throughout to present hypotheses as hypothesis-generating rather than definitive conclusions. Definitive language such as "the critical distinction was" has been replaced with cautious phrasing.

Independent effect of laser therapy: Limitation emphasized consistently throughout Results and Discussion. The Conclusion now explicitly states that observed benefits cannot be attributed to the laser alone.

Dermal remodeling mechanism: Clear distinction maintained between biological rationale and demonstrated therapeutic mechanism. The Introduction now explicitly states that the remodeling hypothesis remains unproven in ADMH.

Disease activity: Presented as a hypothesis for future research requiring prospective evaluation. We explicitly acknowledge that disease activity was not systematically evaluated in the included studies.

Safety and phototypes: Emphasis added on limited representation of Fitzpatrick V–VI. The theoretical safety advantage is now clearly framed as unproven in ADMH.

Editorial revision: Thorough language and terminology review completed throughout the manuscript and supplementary materials.

We believe that these revisions have substantially strengthened the methodological rigor and interpretative clarity of our manuscript. We are grateful for the opportunity to revise and hope that the revised version meets the standards for publication in PLOS One.

We look forward to your further evaluation.

Sincerely,

Virgilio Blandón, MD

Corresponding Author

On behalf of all coauthors

Attachment Submitted filename: Response_to_Reviewers.docx

# Peer review: Decision Letter 1

**DOI:** 10.1371/journal.pone.0359552.r003

**Authors:** Gisele Viana de Oliveira

15 Sep 2026

Non-ablative dermal remodeling lasers in acquired dermal macular hyperpigmentation: A scoping review

PONE-D-26-37100R1

Dear Dr. Blandon,

We’re pleased to inform you that your manuscript has been judged scientifically suitable for publication and will be formally accepted for publication once it meets all outstanding technical requirements.

Within one week, you’ll receive an e-mail detailing the required amendments. When these have been addressed, you’ll receive a formal acceptance letter and your manuscript will be scheduled for publication.

An invoice will be generated when your article is formally accepted. Please note, if your institution has a publishing partnership with PLOS and your article meets the relevant criteria, all or part of your publication costs will be covered. Please make sure your user information is up-to-date by logging into Editorial Manager at Editorial Manager® and clicking the ‘Update My Information' link at the top of the page. For questions related to billing, please contact billing support.

If your institution or institutions have a press office, please notify them about your upcoming paper to help maximize its impact. If they’ll be preparing press materials, please inform our press team as soon as possible -- no later than 48 hours after receiving the formal acceptance. Your manuscript will remain under strict press embargo until 2 pm Eastern Time on the date of publication. For more information, please contact onepress@plos.org.

Kind regards,

Gisele Viana de Oliveira

Academic Editor

PLOS One

Additional Editor Comments (optional):

Thank you for contributing with such an interesting study.

Reviewers' comments:

Reviewer's Responses to Questions

**Comments to the Author**

Reviewer #1: All comments have been addressed

Reviewer #2: All comments have been addressed

**********

2. Is the manuscript technically sound, and do the data support the conclusions?

Reviewer #1: Yes

Reviewer #2: Yes

**********

3. Has the statistical analysis been performed appropriately and rigorously?

Reviewer #1: Yes

Reviewer #2: Yes

**********

4. Have the authors made all data underlying the findings in their manuscript fully available?

The PLOS Data policy

Reviewer #1: Yes

Reviewer #2: Yes

**********

5. Is the manuscript presented in an intelligible fashion and written in standard English?

Reviewer #1: Yes

Reviewer #2: Yes

**********

Reviewer #1: The authors of manuscript PONE-D-26-37100R1, titled "Non-ablative dermal remodeling lasers in acquired dermal macular hyperpigmentation: A scoping review", have satisfactorily and meticulously addressed all comments raised in the previous round of review

The revisions have significantly enhanced the manuscript's balance, methodological transparency, and scientific rigor. By appropriately tempering their clinical claims, acknowledging research limitations, and reconciling all quantitative discrepancies, the authors have produced a highly rigorous scoping review

Detailed Review Comments

1. Technical Soundness and Data Support (PLOS ONE Question 2)

The manuscript describes a technically sound scoping review that follows the Arksey and O'Malley and Levac et al. frameworks and adheres to the PRISMA-ScR guidelines

The authors have resolved previous concerns regarding overclaiming clinical efficacy:

Softened Causal Interpretations: The discrepancy between negative randomized controlled trials (RCTs) and positive observational reports is now correctly framed as hypothesis-generating rather than as a definitive causal conclusion

The authors have replaced definitive language (such as "the critical distinction was") with cautious phrasing, noting that factors like continuous topical tacrolimus and intervention during stable disease phases are interesting biological hypotheses requiring prospective validation

Independent Laser Efficacy: The authors have consistently highlighted that because the vast majority of favorable observational studies utilized concomitant therapies (such as tacrolimus, systemic corticosteroids, cysteamine, PDRN, or multimodal protocols), the independent therapeutic contribution of non-ablative dermal remodeling lasers (NADRL) cannot be established from the current evidence

Dermal Remodeling Mechanism: The manuscript now clearly distinguishes between the biological rationale for laser use (dermal remodeling) and the clinical endpoints actually measured (pigmentation)

The authors have openly acknowledged that the remodeling hypothesis remains unproven in ADMH and have added constructive recommendations for future studies to incorporate objective structural, histological, and molecular markers

Disease Activity and Phototype Safety: The revised text explicitly notes that disease activity was not systematically defined or prospectively evaluated in the included literature

Furthermore, the safety profile of these lasers in darker skin types has been balanced by emphasizing that Fitzpatrick skin types V–VI are severely underrepresented (only one study included a single Fitzpatrick V patient and none included Fitzpatrick VI), meaning that theoretical safety advantages of water-targeting lasers remain unproven in these higher-risk populations

2. Statistical and Quantitative Rigor (PLOS ONE Question 3)

The quantitative metrics and study-selection numbers have been completely reconciled and explained consistently across the Abstract, Results, and PRISMA-ScR flow diagram:

1,267 total records were retrieved from all information sources

193 records were identified through electronic database searches and carried forward for formal screening and deduplication

1,074 records were identified through citation tracking of reviews and included studies, none of which progressed past pre-screening as they did not meet eligibility criteria

7 studies were included in the final review

31 unique patients are synthesized in the review

The authors correctly accounted for a subset of six patients with ashy dermatosis shared between the two trials by Kroon et al. and Wind et al., ensuring that the final population is not misreported as 37

3. Data Availability (PLOS ONE Question 4)

The authors have complied fully with the PLOS Data policy

All search strategies, screening decisions, exclusion codes, data extraction forms, risk of bias assessments, and the completed PRISMA-ScR checklist have been made fully available as supporting information (S1–S5 Tables, S1–S2 Texts, and S1 Appendix)

4. Language and Presentation (PLOS ONE Question 5)

The manuscript is intelligible, written in clear, standard English, and presents a logical flow

The authors completed a thorough language and terminology review to correct typographical inconsistencies and standardize all abbreviations (such as ADMH, NADRL, DPASI, and PIH) throughout both the manuscript and its supplementary files

5. Ethical Concerns and Competing Interests

The scoping review correctly states "N/A" for its ethics statement since it synthesizes previously published primary literature and did not involve direct human or animal research

Funding declarations are transparent (the study received no external funding)

The authors have declared no conflicts of interest, with appropriate disclosure regarding industry-affiliated advisory boards for specific devices which had no role in study design or data interpretation

Reviewer #2: The authors have adequately addressed the comments raised in the previous review. I have no further comments.

**********

what does this mean?). If published, this will include your full peer review and any attached files.

If you choose “no”, your identity will remain anonymous but your review may still be made public.

**Do you want your identity to be public for this peer review?**  For information about this choice, including consent withdrawal, please see our Privacy Policy

Reviewer #1: **Yes:**  Patricia Apt Druck

Reviewer #2: No

**********

# Editor report: Acceptance letter

**DOI:** 10.1371/journal.pone.0359552.r004

**Authors:** Gisele Viana de Oliveira

PONE-D-26-37100R1

PLOS One

Dear Dr. Blandon,

I'm pleased to inform you that your manuscript has been deemed suitable for publication in PLOS One. Congratulations! Your manuscript is now being handed over to our production team.

At this stage, our production department will prepare your paper for publication. This includes ensuring the following:

* All references, tables, and figures are properly cited

* All relevant supporting information is included in the manuscript submission,

* There are no issues that prevent the paper from being properly typeset

You will receive further instructions from the production team, including instructions on how to review your proof when it is ready. Please keep in mind that we are working through a large volume of accepted articles, so please give us a few days to review your paper and let you know the next and final steps.

Lastly, if your institution or institutions have a press office, please let them know about your upcoming paper now to help maximize its impact. If they'll be preparing press materials, please inform our press team within the next 48 hours. Your manuscript will remain under strict press embargo until 2 pm Eastern Time on the date of publication. For more information, please contact onepress@plos.org.

You will receive an invoice from PLOS for your publication fee after your manuscript has reached the completed accept phase. If you receive an email requesting payment before acceptance or for any other service, this may be a phishing scheme. Learn how to identify phishing emails and protect your accounts at https://explore.plos.org/phishing.

If we can help with anything else, please email us at customercare@plos.org.

Thank you for submitting your work to PLOS One and supporting open access.

Kind regards,

PLOS One Editorial Office Staff

on behalf of

Dr. Gisele Viana de Oliveira

Academic Editor

PLOS One
